# Restorative reproductive medicine for infertility in two family medicine clinics in New England, an observational study

**DOI:** 10.1186/s12884-021-03946-8

**Published:** 2021-07-07

**Authors:** Joseph B. Stanford, Paul A. Carpentier, Barbara L. Meier, Mark Rollo, Benjamin Tingey

**Affiliations:** 1 Office of Cooperative Reproductive Health, Department of Family and Preventive Medicine, University of Utah School of Medicine, 375 Chipeta Way, Suite A, Salt Lake City, UT 84108 USA; 2 International Institute for Restorative Reproductive Medicine, London, UK; 3 Gianna of Long Island Center for Women’s Health and Fertility, New York, USA; 4 In His Image Family Medicine, Gardner, MA USA; 5 Reliant Medical Group, Fitchburg, MA USA; 6 Department of Family and Preventive Medicine, University of Utah School of Medicine, Salt Lake City, UT USA

**Keywords:** Infertility, Restorative reproductive medicine, Infertility, treatment, Infertility, etiology, Infertility, treatment outcomes

## Abstract

**Background:**

Restorative reproductive medicine (RRM) seeks to identify and correct underlying causes and factors contributing to infertility and reproductive dysfunction. Many components of RRM are highly suitable for primary care practice. We studied the outcomes amongst couples who received restorative reproductive medicine treatment for infertility in a primary care setting.

**Methods:**

Two family physicians in Massachusetts trained in a systematic approach to RRM (natural procreative technology, or NaProTechnology) treated couples with infertility. We retrospectively reviewed the characteristics, diagnoses, treatments, and outcomes for all couples treated during the years 1989 to 2014. We compared pregnancy and live birth by clinical characteristics using Kaplan-Meier analysis. We employed the Fleming-Harrington weighted Renyi test or the logrank test to compare the cumulative proportion with pregnancy or with live birth.

**Results:**

Among 370 couples beginning treatment for infertility, the mean age was 34.8 years, the mean prior time trying to conceive was 2.7 years, and 27% had a prior live birth. The mean number of diagnoses per couple was 4.9. Treatment components included fertility tracking with the Creighton Model FertilityCare System (80%); medications to enhance cervical mucus production (81%), to stimulate ovulation (62%), or to support the luteal phase (75%); and referral to female laparoscopy by a surgeon specializing in endometriosis (46%). The cumulative live birth rate at 2 years was 29% overall; this was significantly higher for women under age 35 (34%), and for women with body mass index < 25 (40%). There were 2 sets of twins and no higher-order multiple gestations. Of the 63 births with data available, 58 (92%) occurred at term.

**Conclusions:**

Family physicians can provide a RRM approach for infertility to identify underlying causes and promote healthy term live births. Younger women and women with body mass index < 25 are more likely to have a live birth.

**Supplementary Information:**

The online version contains supplementary material available at 10.1186/s12884-021-03946-8.

## Background

Infertility is a common concern in couples [1, 2]. It is not only associated with increasing age, but can be caused by many underlying pathophysiologic mechanisms in women and/or men [1, 3]. Improved understanding of these mechanisms and their diagnosis and treatment could improve obstetrical outcomes and long-term health of the parents and offspring [4], and generate significant savings for the cost of fertility treatment [5]. Primary care physicians, and family physicians in particular, can serve an important role for infertility evaluation and treatment because infertility 1) is common; 2) is a couple’s issue; 3) involves coincident chronic disorders impacting fertility that can be addressed in primary care [4, 6]. Initial management of infertility by primary care specialists with subsequent referral as needed can result in similar time to pregnancy as initial management by fertility subspecialists [7].

Treatment strategies for infertility include those that accomplish some parts of the reproductive process outside of the body (assisted reproductive technology, ART), and those that seek exclusively to restore normal physiologic fertility (restorative reproductive medicine, RRM). Assisted reproductive technology techniques include in vitro fertilization (IVF), with or without intracytoplasmic sperm injection (ICSI), and intrauterine insemination [8]. RRM includes lifestyle changes to improve health and reproductive function, educating women/couples to understand their fertility cycle and the fertile window, medical treatments supporting ovulation, implantation, immune function, spermatogenesis, and other physiologic processes related to fertility, and surgery to remove pathologic tissue and restore normal anatomy and function [9]. Central to the RRM approach is seeking to identify underlying causes or contributing factors [10, 11].

A specific model of RRM is called natural procreative technology (also known as NaProTechnology), developed at Creighton University School of Medicine and the Saint Paul VI Institute for the Study of Human Reproduction. It includes a standardized system for educating couples about the fertility cycle, called the Creighton Model Fertility Care System (Creighton Model), and medical and surgical treatments to support conception in vivo [10, 12, 13]. Several studies have been published regarding the NPT treatment of infertility; however, additional data are needed to assess outcomes in different settings and the impact of clinical factors on outcomes [9, 14–16].

This paper presents results of a retrospective cohort study of RRM of all infertile couples referred to two primary care practices for evaluation and treatment. The primary outcomes are the cumulative proportion of couples experiencing conception and live births. The secondary outcomes are preterm birth and low birth weight. We assessed the impact of demographic and clinical characteristics on the primary outcomes. We also characterized the processes of care by evaluating the diagnoses and the treatments administered.

## Methods

In this retrospective observational study, we analyzed all infertility patients evaluated and treated by 2 family physicians in separate independent practices in Massachusetts between 1989 and 2014. Both physicians are formally trained and certified in NPT. Patients were received predominantly by referral from other physicians and fertility educators or lay referral, and were usually seeking fertility treatment not involving ART, for various reasons, including personal and religious values, or cost. Criteria for patient inclusion were at least 1 office visit during the study period; at least one lab evaluation related to fertility; the absence of clinical pregnancy despite at least 1 year (or in women age ≥ 35 years, at least 6 months) trying to conceive [17]. Time trying to conceive started at the couple’s reported first month of sexual intercourse without methods to avoid pregnancy, or the conclusion of their last pregnancy (often a miscarriage), whichever came last. Couples were considered to have started RRM treatment at the date of first clinic consult related to fertility evaluation, or the date they had been trying to conceive for 1 year (or 6 months for women with age ≥ 35 years), whichever came later.

The procedures of medical NPT used were similar to those reported previously [15, 16, 18]. The initial evaluation for each patient included teaching the couple to track ovulation and other menstrual cycle parameters (usually with the Creighton Model); an initial medical history (both partners) and physical exam (always the woman and sometimes the man); pre-ovulatory and mid luteal–targeted hormonal testing. If endometriosis or surgically correctable conditions were suspected, additional evaluations such as pelvic ultrasound, hysterosalpingography, and referral for laparoscopy were arranged. Semen analysis was recommended routinely, but not always completed. Based on results of these evaluations, appropriate diagnoses were made for underlying and related conditions.

Treatments were prescribed to restore or optimize normal reproductive physiology to the extent possible, i.e., to assure regular ovulation, appropriate cervical mucus production, optimal timing of intercourse, and appropriate luteal phase hormonal function. Patients were encouraged to maximize preconception health, including appropriate weight loss, and treated any underlying condition that might contribute to impaired fertility, implantation, or successful pregnancy.

Data were collected via review of medical records. These included patient characteristics, diagnoses, treatments employed, pregnancy, live births, number of fetuses, birth weight and duration of pregnancy. To ascertain pregnancy outcomes, patients were contacted, when possible, via mail and telephone. We used partially de-identified data for this analysis. Each physician obtained local Institutional Review Board approval, and the study was also approved by the Institutional Review Board at the University of Utah.

We calculated descriptive statistics for all eligible patients. We compared specific fertility diagnoses before and after NPT evaluation using McNemar’s test statistic. We calculated frequencies of treatments received, crude proportions of couples conceiving or having a live birth over 2 years, and Kaplan-Meier survival curves to adjust for dropout from treatment. We conducted stratified analyses by clinical factors that we expected to impact the likelihood of pregnancy and birth, with the following factors chosen a priori, based on existing literature: woman’s age, time trying to conceive, prior pregnancy, prior live birth, prior IVF, prior intrauterine insemination (IUI). We also subsequently evaluated the impact of body mass index (BMI) and the treatment start date on the primary outcomes [15, 19, 20]. For most stratified analyses, the survival curves crossed, and we employed the Fleming-Harrington weighted Renyi test to compare the cumulative proportion with pregnancy or with live birth. For survival analyses where the survival curves did not cross, we employed the longrank test. The proportions of births with multiple gestation, low birth weight, and prematurity were calculated. Because this was a descriptive analysis of outcomes from all eligible patients, we did not conduct sample size or power calculations.

## Results

Between 1989 and 2014, 559 patients were evaluated for fertility concerns. After excluding couples who did not meet criteria or who had missing data, there were 370 eligible couples. Half of eligible women were age 35 or older, 46% had experienced a prior pregnancy, and 27% had a previous live birth. The mean time trying to conceive prior to entry was 2.7 years. Additional characteristics of the couples are given in Table 1.

**Table 1:** Characteristics of Subfertile Couples Beginning Treatment with Natural Procreative Technology (n=370)

Patient Characteristic	n (%)
Woman’s age, mean (SD) [minimum-maximum], y	34.8 (5.86) [21-49]
≥35	186 (50)
Time attempting to conceive, mean (SD) [minimum-maximum], y	2.67 (3) [0.5-19.6]
<1	97 (26)
1-2.9	165 (45)
≥3	108 (29)
BMI, mean (SD) [minimum-maximum]^a^	25.58 (6.15) [17-51]
<25	154 (56)
≥25	121 (44)
Had prior pregnancy	169 (46)
Had prior live birth^a^	99 (27)
Had prior miscarriage	118 (32)
Had 3 or more prior miscarriages	22 (6)
Received prior in vitro fertilization^a^	21 (6)
Received prior intrauterine insemination	49 (13)
Patients of Dr. Carpentier	316 (85)
Patients of Dr. Rollo	54 (15)

*BMI* body mass index, *IUI* intrauterine insemination, *IVF in vitro* fertilization. *SD* standard deviation

^a^Missing data as follows: BMI=95; prior live birth=2; IVF=1

The mean number of fertility-related diagnoses per couple after evaluation was 4.9 (range, 0–14). The most common diagnoses were endometriosis (74%), limited cervical mucus (65%), and ovarian dysfunction identified based on hormonal profiles (66%), the majority of which had a component of low luteal progesterone (56%). Male factor was diagnosed in 30% of couples. A female mental health diagnosis (primarily depression) was identified in 25% of couples. Details of diagnoses are given in Table 2.

**Table 2:** Diagnoses Among Infertile Couples Before and After Natural Procreative Technology Evaluation (n=370)^a^

Diagnostic Category	Before NPT Evaluation, n (%)	After NPT Evaluation, n (%)	***P*** Value
Unexplained infertility	86 (23)	2 (1)	<.0001
Pregnant before evaluation completed	1 (0)	NA	NA
Male factor	36 (10)	110 (30)	<.0001
Endometriosis	50 (14)	275 (74)	<.0001
Blocked fallopian tubes	18 (5)	56 (15)	<.0001
Pelvic adhesions	16 (4)	89 (24)	<.0001
Polycystic ovarian syndrome	34 (9)	73 (20)	<.0001
Ovarian dysfunction	NA	246 (66)	NA
Anovulation	4 (1)	25 (7)	<.0001
Low periovulatory estrogen	NA	130 (35)	NA
Low luteal estrogen	1 (0)	60 (16)	<.0001
Low luteal progesterone	14 (4)	208 (56)	<.0001
Limited cervical mucus	7 (2)	241 (65)	<.0001
Hypothyroidism	24 (6)	37 (10)	0.037
Fibroids	21 (6)	32 (9)	0.048
Premenstrual syndrome	NA	161 (44)	NA
Abnormal vaginal bleeding	NA	70 (19)	NA
Mental health diagnosis, female^b^	NA	93 (25)	NA
Diminished ovarian reserve	NA	45 (12)	NA
Sexual dysfunction, female or male	NA	46 (13)	NA
Elevated prolactin in female	NA	18 (5)	NA
Vitamin D deficiency	NA	53 (14)	NA

*NA* not applicable or not available, *NPT* Natural Procreative Technology

^a^Most couples had multiple diagnoses (mean number of diagnoses, 4.9; SD, 2.3; range, 0-14)

^b^Primarily depression

The median number of office visits per couple was 4 (range, 1–22). The large majority (80%) tracked ovulation and the fertile days with the Creighton Model [12], while 14% used other systems of tracking fertility (primarily the Sympto-Thermal method) [21–23]. Nearly half the records (44%) had lifestyle advice documented. Almost all (96%) received medical treatment, including medications to enhance mucus production (81%), clomiphene (30%), letrozole (48%), luteal progesterone (73%) or luteal human chorionic gonadotropin (15%). Additional details of treatments are noted in Table 3.

**Table 3:** Treatments for Infertile Couples (n=370)

Treatment	n (%)
Number of office visits	
Mean	4.6
Median	4
[minimum-maximum]	[1-22]
Number of coordinated cycles of treatment^a^	
Mean	12.3
Median	10
[minimum-maximum]	[1-80]
Type of fertility cycle tracking	
Creighton Model	297 (80)
Other^b^	51 (14)
None	22 (6)
Vitamins and supplements	302 (82)
Folic acid	231 (63)
Vitamin D	202 (55)
Magnesium	160 (43)
Pycnogenol	63 (17)
Iodine	58 (16)
Probiotic	19 (5)
Iron	21 (6)
Vitamin E	15 (4)
Avoid Vitamin C	80 (22)
Miscellaneous supplements	71 (19)
Lifestyle advice	164 (44)
Advice for female weight loss	56 (15)
Advice for female weight gain	14 (4)
Any other advice about diet or exercise	126 (34)
Advice about sleep	67 (18)
Advice about stress management	74 (20)
Avoid chemical exposures	33 (9)
Any medical treatments	356 (96)
Medications to enhance cervical mucus production^c^	299 (81)
Any ovulation drug	229 (62)
Clomiphene	111 (30)
Letrozole	176 (48)
Injectable ovulation drug	9 (2)
Drugs influencing insulin/glucose metabolism (primarily metformin)	85 (23)
Any luteal hormonal support	279 (75)
Luteal progesterone	267 (73)
Luteal human chorionic gonadotropin	54 (15)
Low-dose naltrexone	164 (44)
Thyroid hormone supplementation	39 (11)
Piroxicam for 3 days prior to the predicted time of implantation	73 (20)
Antidepressant	34 (9)
Antibiotics for infection	13 (4)
Advice to discontinue antihistamines	19 (5)
Other medications	33 (9)
Surgeries, women	176 (48)
Laparoscopy^d^	169 (46)
Other female surgery	15 (4)
Any male treatment^e^	81 (22)

^a^Missing data for 4 women

^b^Includes Sympto-Thermal (n=56), Billings ovulation method (n=2), Marquette model (n=1)

^c^Includes vitamin B6, guaifenesin, amoxicillin, cephalexin, erythromycin

^d^By referral to surgeon. Often, laparoscopy revealed endometriosis, which was usually treated by excision or ablation. In some cases, laparoscopy also involved other interventions, such as lysis of adhesions or ovarian drilling

^e^Includes lifestyle advice, antioxidant and other supplements, antibiotics, clomiphene, sildenafil, referral for varicocele surgery

**Fig. 1:**
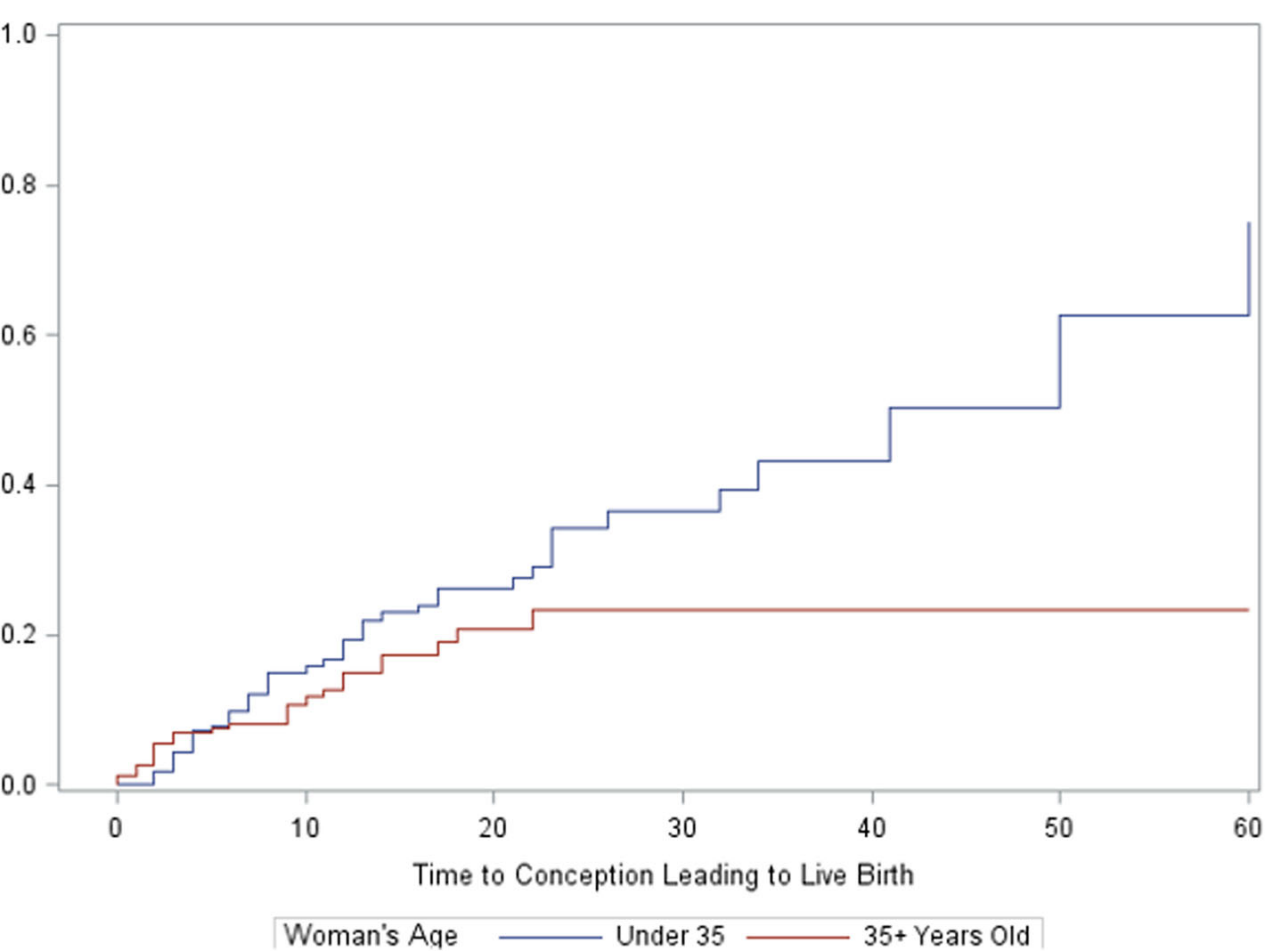
Cumulative probability of conception resulting in live birth by woman’s age at entry to treatment (Kaplan-Meier curves)

**Fig. 2:**
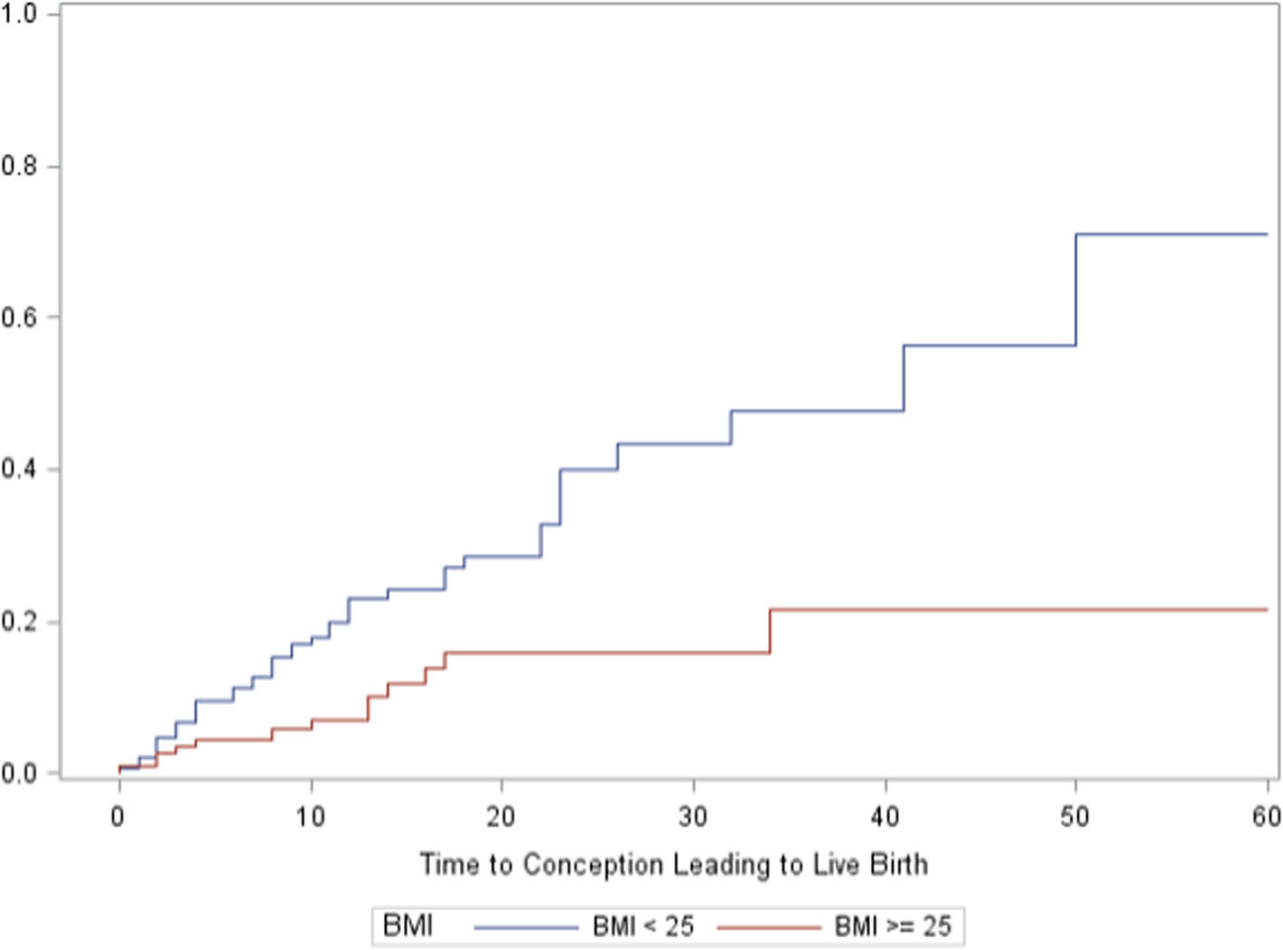
Cumulative probability of conception resulting in live birth by body mass index (kg/m^2^)

The unadjusted proportion with a pregnancy and live birth were 31 and 18%. Adjusting for dropout with Kaplan-Meier analyses, the proportions were 39 and 29%, respectively (Table 4). Dropout before 2 years of treatment or pregnancy was 56% overall. Characteristics associated with a significantly higher adjusted proportion of live birth included woman’s age < 35 years (34%), women age > 34 trying less than 1 year (38%), and woman’s BMI < 25 (40%) (Table 4 and Figs. 1 and 2, with further detailed figures in Appendix). There were too few patients with BMI < 18.5 to analyze separately. There was no statistically significant difference in live birth rates by gravidity, parity, or prior IVF or IUI treatment (summarized in Table 4, with detailed figures in Appendix). Women who tracked ovulation and fertile days with the Creighton Model, other fertility charting, or no charting had a cumulative adjusted proportion with live births of 30, 30, and 10%, respectively; a difference that was not statistically significant. The Kaplan-Meier curves for these latter characteristics are presented in the Appendix.

**Table 4:** Discontinuations, Conceptions, and Conceptions Leading to Live Births up to 24 Months After Beginning Natural Procreative Technology Treatment, by Characteristics of Couples Beginning Treatment

Couple Characteristics	Couples n	Exited Treatment, n (%)	Conception, n (%)	Live Births, n (%)	Adjusted Conceptions, %	***P*** value	Adjusted Live Births, %	***P*** value
All couples	370	209 (56)	116 (31)	66 (18)	39		29	
Age, y						0.0216^a^		0.0438^a^
<35	184	91 (49)	64 (35)	40 (22)	44		34	
≥35	186	118(63)	52 (28)	26 (14)	33		23	
Time trying for birth, y						<0.001^a^		<0.001^a^
<1	97	45 (46)	40 (41)	24 (25)	52		38	
1-2.9	165	90 (55)	58 (35)	33 (20)	42		33	
≥3	108	74 (69)	18 (17)	9 (8)	15		12	
Had prior pregnancy						0.0129^a^		0.1299^a^
Yes	169	92(54)	66(39)	35 (21)	52		36	
No	201	117 (58)	50 (25)	31 (15)	29		25	
Had prior live birth						0.1040^a^		0.6249^a^
Yes	99	58 (59)	34 (34)	18 (18)	54		35	
No	269	150 (56)	82 (30)	48 (18)	34		28	
Received prior IVF						0.1861^**b**^		0.7490^a^
Yes	21	16 (76)	4 (19)	2 (10)	30		20	
No	348	192 (55)	112 (32)	64 (18)	40		30	
Received prior IUI						0.2305^a^		0.1836^a^
Yes	49	33 (67)	11 (22)	5 (10)	21		15	
No	320	175 (55)	105 (33)	61 (19)	41		32	
Physician						0.7869^a^		0.7506^a^
Dr. Carpentier	316	179 (57)	98 (31)	56 (18)	39		30	
Dr. Rollo	54	30 (56)	18 (33)	10 (19)	40		27	
Menstrual/fertility cycle charting						0.7551^a^		0.5753^a^
Creighton Model	297	167 (56)	92 (31)	53 (18)	38		30	
Other	51	25 (49)	18 (35)	12 (24)	42		30	
None/missing	22	17 (77)	6 (27)	1 (2)	49		10	
BMI						0.0812^**b**^		0.0008^**b**^
<25	154	83 (54)	54 (35)	38 (25)	45		40	
≥25	121	65 (54)	34 (28)	12 (10)	31		16	
Start date (tertiles)						0.0850^a^		0.8055^a^
Dec 1990-June 2005	113	72 (64)	30 (27)	17 (15)	33		25	
June 2005-Mar 2010	130	58 (45)	44 (34)	31 (24)	42		34	
Mar 2010- Dec 2013	126	79 (63)	41 (33)	18 (14)	38		28	

*BMI* body mass index, *IUI* intrauterine insemination, *IVF in vitro* fertilization

^a^Fleming-Harrington weighted Renyi test

^b^Adjusted log-rank test

For live births conceived with NPT (*n* = 68 from 66 pregnancies), 58 (92%) were born at term; 5 (8%) at 32 to 37 weeks gestation; 3 had missing data. There were only 2 sets of twins, and no higher-order multiple births (details in Table A-1, Appendix).

## Discussion

Restorative reproductive medicine provided by two NPT-trained family physicians in separate practices in New England yielded an overall adjusted cumulative live birth proportion of 29%. The birth proportion was significantly higher for women < 35 years of age, those trying less than 1 year at entry (who were, by definition, all 35 years of age or older and had been trying for at least 6 months), and for those with BMI < 25. There were 2 sets of twins and no higher-order multiple births.

### Strengths

Our study adds to the growing literature supporting an integrated RRM approach to infertility. It suggests that the RRM approach in a primary care setting can identify underlying causes or contributing factors for infertility and that treatment results in healthy births for a significant proportion of couples.

This is the first study that has examined the impact of woman’s BMI within RRM, which we investigated as a secondary analysis. Women who had BMI < 25 had 40% probability of live birth, compared to 16% probability for women with BMI ≥25. This is concordant with studies showing lower live birth rates among infertile women with a high BMI who undergo other types of fertility treatment, including intrauterine insemination, IVF, or simply ovulation induction [24–26]. Future studies of RRM and all fertility treatments should continue to examine this important risk factor.

Women > 35 years of age who had been trying from 6 months to 1 year based on standard clinical definitions and recommendations [17], had substantially higher rates of pregnancy leading to live birth than all women who were trying for more than 1 year. Because we focused on infertility, we did not include women under 35 years of age trying for less than one year. Regardless of age, we believe it is reasonable that all women with greater than 6 months of trying begin tracking their fertility cycles and consider RRM evaluation to facilitate evaluation and achieving healthy pregnancy more quickly.

### Limitations

Without a control group we cannot identify the untreated spontaneous birth rate, which limits the ability to infer the impact of treatment. Because the patients were received predominantly by referral rather than being population-based, we believe a spontaneous birth rate for a referral population is a more relevant comparison than one for a population-based primary care practice; the former is about 50% lower than the latter [27]. The proportion with live birth (10%) among those who did no fertility charting might be one surrogate comparison for a minimal intervention group, but the number of couples in this group was small (22 couples). Future studies should seek more robust comparison groups, possibly of different treatments, because couples seeking medical attention for infertility are usually not willing to go without any treatment.

The diagnoses prior to RRM evaluation (Table 2) were reported by patients (who may not remember all diagnoses they were given) or sometimes available from prior medical records. Diagnostic criteria and the intensity of diagnostic evaluation will vary between different practices, and the patients came from many prior practices. Therefore, the comparisons between diagnoses before and after evaluation should be considered as descriptive and perhaps suggestive, and certainly not definitive.

Although all patients received RRM evaluation, not all couples availed themselves of fertility tracking. Creighton Model Fertility Care System tracking is the foundation of NPT [13, 28, 29]. Most study patients (80%) used the Creighton Model. However, 14% of couples used other types of fertility tracking, primarily the Sympto-Thermal method, which tracks cervical fluid, bleeding, and basal body temperature [22, 23]. We did not find a difference in proportions of live births between these 3 groups, (30% vs. 30%, respectively). Further research is needed to define the potential impact of different types of fertility tracking [30, 31].

Encouraging infertile couples to continue for a full trial of treatment represents a challenge. Over half of our study couples discontinued treatment before 2 years. This rate of discontinuation is similar for that of other infertility treatment cohorts, both RRM and ART [9, 15, 16, 32, 33].

### Comparisons to prior RRM studies

These are the first published data on RRM for infertility from family physicians in the United States, and complements previously published data from family physicians in Ireland and Canada. The cumulative adjusted proportion of live births in those studies was 53% in Ireland (32% for a separate study of only couples who had previous IVF), and 66% in Canada [9, 15, 16]. The reported risk factors measured in the Canadian study were similar to those reported here, and the reasons for the lower proportion with live birth in the present study are unclear. One possible difference between populations could be the women’s BMI, which was not reported in the Irish or Canadian studies. There is also variation in interventions. For example, in the Irish group, follicular ultrasound tracking was used routinely, whereas it was rarely used in these New England practices. This may correlate with a less aggressive approach to ovulation stimulation in the patients in this study.

Another difference relates to inclusion criteria. The criteria for this study were intentionally broad (only 1 visit and 1 lab test). In both the Irish and Canadian studies, at least 2 clinic visits were required for inclusion. The survival analysis should adjust for early drop out, but if those who dropped out after one visit from the present study had a lower potential for pregnancy than those who continued for two visits or more, this could contribute to a lower cumulative pregnancy probability in the present study, compared to studies that required two visits to be entered in the study, and thus excluded the couples who had only one visit. Finally, differences in the duration of follow-up may contribute to differences in survival analysis probabilities.

A broad range of interventions was used in these patients (see Table 3). A review of the available evidence for each specific intervention to improve normal reproductive function is beyond the scope of this paper. We also lack adequate timing data to construct the statistical models to identify treatments that may be most successful for couples with various underlying diagnoses. These issues should be addressed in future and larger prospective studies.

### Birth outcomes

The proportions of births with prematurity (8%) and low birth weight (2%) were very low, and compared very favorably to the recent Massachusetts state average prematurity rate of 8.6% in 2014 [34]. This supports the concept that RRM treatment, by identifying and rectifying underlying chronic disease processes, can result in better maternal and newborn outcomes compared to ART. Even when comparison is restricted to singleton births, IVF in the U.S. was associated with rates of prematurity and low birth weight of 30.9 and 26.7%; and artificial insemination with rates of 15.9 and 12.2%, respectively [35]. One of the fundamental principles of RRM is that value is added to the treatment process by increasing the probability of a healthy pregnancy and neonate [9, 10, 15, 16, 36]. This study gives insight into common underlying conditions that are diagnosed and treated with an RRM approach. We suggest that for best outcomes for the woman, the couple, and the newborn, infertility should be approached as a symptom resulting from multiple, identifiable, chronic underlying causes. This perspective is well suited for primary care settings. Another advantage of RRM is lower cost, particularly relative to IVF. Future cost-effectiveness analyses should include the costs of prenatal, perinatal, neonatal and pediatric care. It is important to study the long-term outcomes of these techniques to ascertain whether RRM treatment leads to better future health for women, men, and their children, and perhaps also lower healthcare costs.

## Conclusion

Family physicians can provide a RRM approach for infertility to identify underlying causes and promote healthy term live births. Younger women, women age > 34 trying less than 1 year, and women with body mass index < 25 are more likely to have a live birth.

## Supplementary Information

**Additional file 1.**

## Data Availability

The de-identified data sets used and / or analyzed during the current study are archived at the University of Utah School of Medicine, Department of Family and Preventive Medicine, Office of Cooperative Reproductive Health. They are available from the corresponding author on reasonable request, subject to IRB review.

## Abbreviations

ART: Assisted reproductive technology
RRM: Restorative reproductive medicine
IVF: In vitro fertilization
IUI: Intrauterine insemination
ICSI: Intracytoplasmic sperm injection
NaProTechnology, NPT: Natural procreative technology
BMI: Body mass index
