# Supplementary material for: Restorative reproductive medicine for infertility in two family medicine clinics in New England, an observational study
Source: BMC Pregnancy Childbirth. 2021 Jul 7;21:495. doi: 10.1186/s12884-021-03946-8 (PMC8265110; doi:10.1186/s12884-021-03946-8)

**Table A-1**. Outcomes for live births conceived with Natural Procreative Technology (n=68 newborns from 66 live births)

| **Outcome** | **n (%)** |
| --- | --- |
| Multiple gestation |  |
| Twins | 2 (3) |
| Triplets or more | 0 (0) |
| Gestational age, weeks^a^ |  |
| ≥37 | 58 (92) |
| 32 to <37 | 5 (8) |
| <32 | 0 (0) |
| Missing | 3 (NA) |
| Birth weight, g^b^ |  |
| ≥2500 | 42 (95) |
| 1501-2499 | 2 (5) |
| ≤1500 | 0 (0) |
| Missing | 24 (NA) |

^a^Multiple births are not included for gestational age (n=66).

^b^Multiple births are included for birth weight (n=68).

**All Figures give Kaplan-Meier curves that are summarized in Table 4.**

**Figure S-1.** **Cumulative probability of conception by woman’s age at entry to NPT treatment. (Kaplan-Meier curves)**


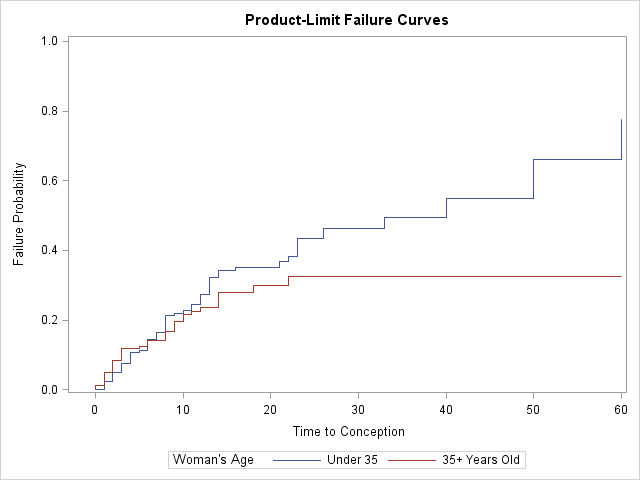


**Figure S-2.** **Cumulative probability of conception by time trying to conceive at entry to NPT treatment. (Kaplan-Meier curves)***


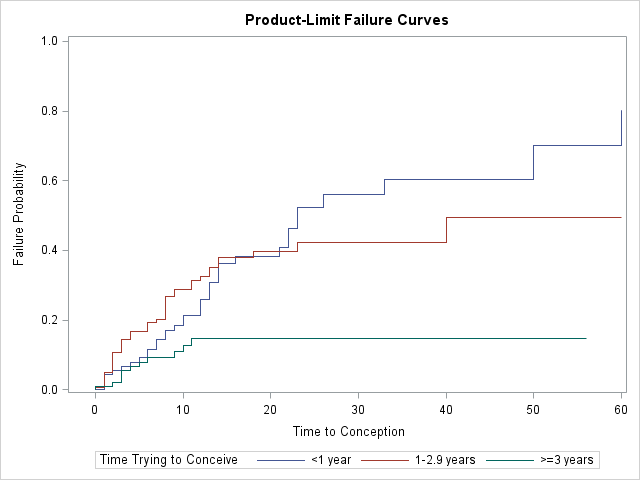


**Figure S-3.** **Cumulative probability of conception resulting in live birth by time trying to conceive at entry to NPT treatment. (Kaplan-Meier curves)***


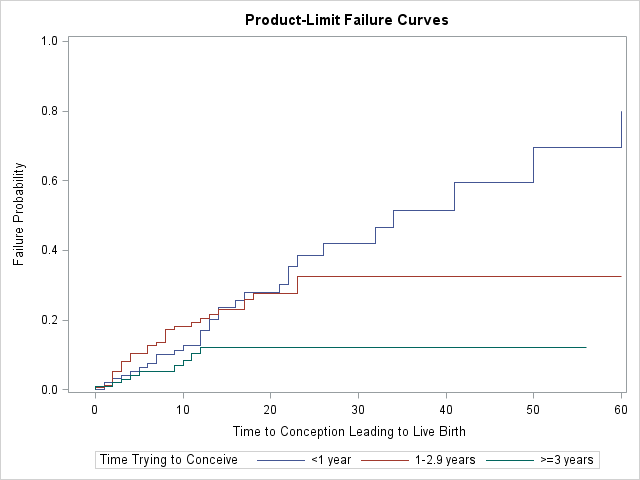


**Figure S-4.** **Cumulative probability of conception by woman’s gravidity prior to entry to NPT treatment. (Kaplan-Meier curves)***


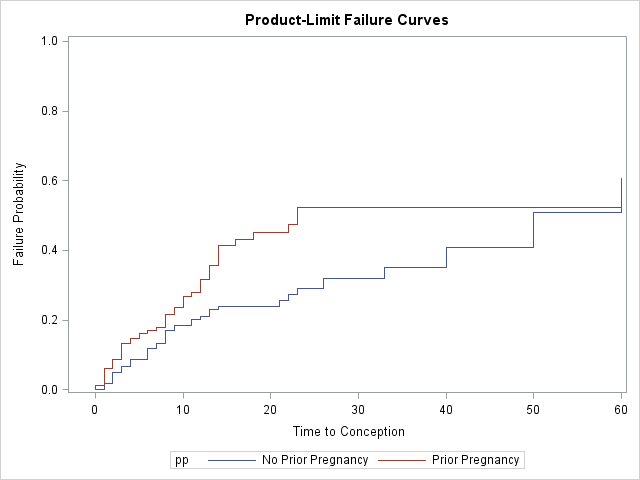


**Figure S-5.** **Cumulative probability of conception leading to a live birth by woman’s gravidity prior to entry to NPT treatment. (Kaplan-Meier curves)**


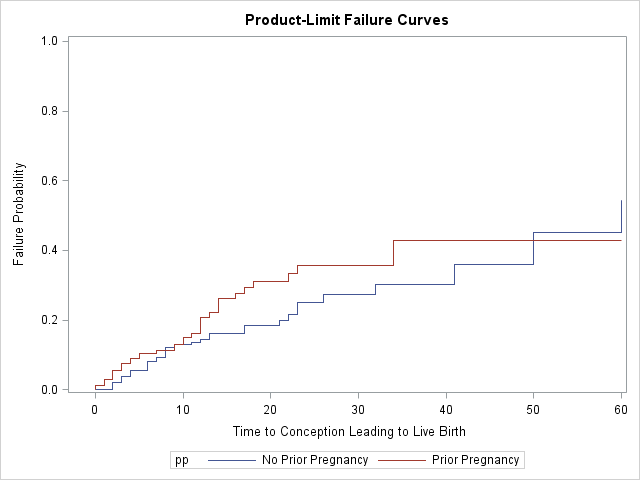


**Figure S-6.** **Cumulative probability of conception by woman’s parity prior to entry to NPT treatment. (Kaplan-Meier curves)**


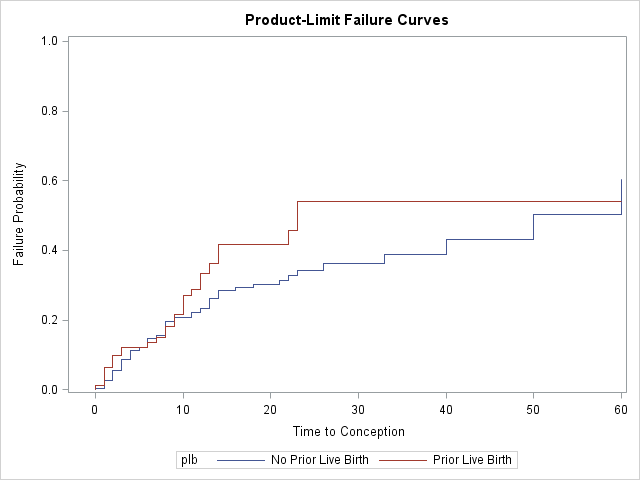


**Figure S-7.** **Cumulative probability of conception resulting in live birth by woman’s parity prior to entry to NPT treatment. (Kaplan-Meier curves)**


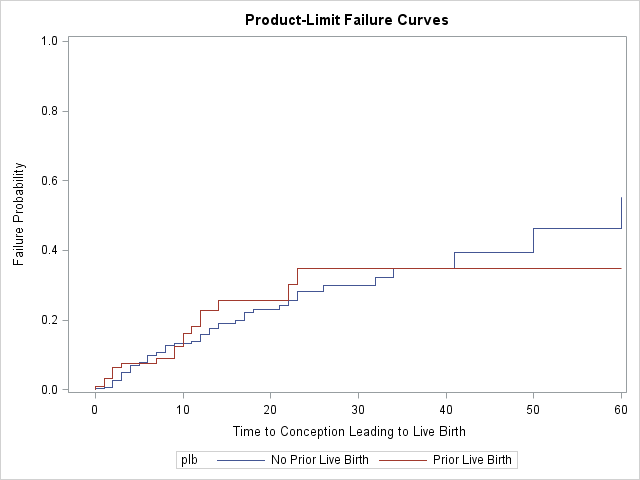


**Figure S-8.** **Cumulative probability of conception by whether the woman previously received treatment with in vitro fertilization. (Kaplan-Meier curves)**


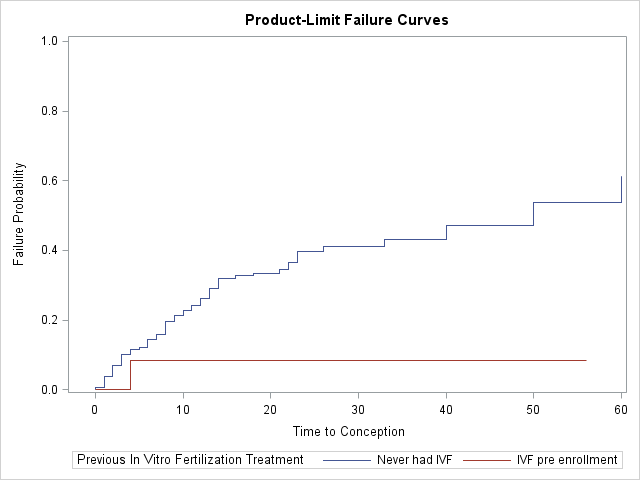


**Figure S-9.** **Cumulative probability of conception resulting in live birth by whether the woman previously received treatment with in vitro fertilization. (Kaplan-Meier curves)**


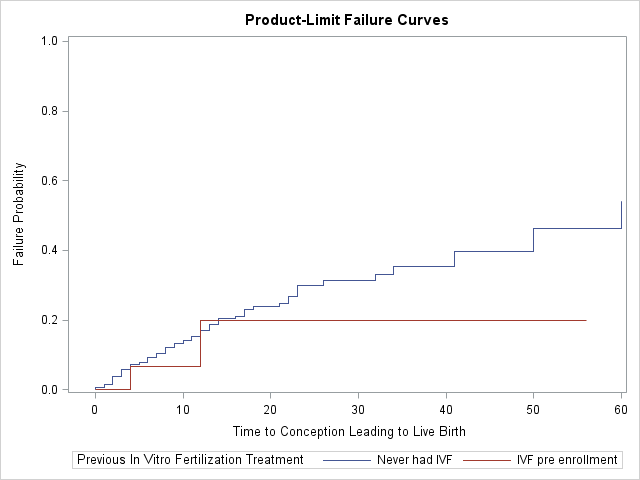


**Figure S-10.** **Cumulative probability of conception by whether the woman previously received treatment with intrauterine insemination. (Kaplan-Meier curves)**


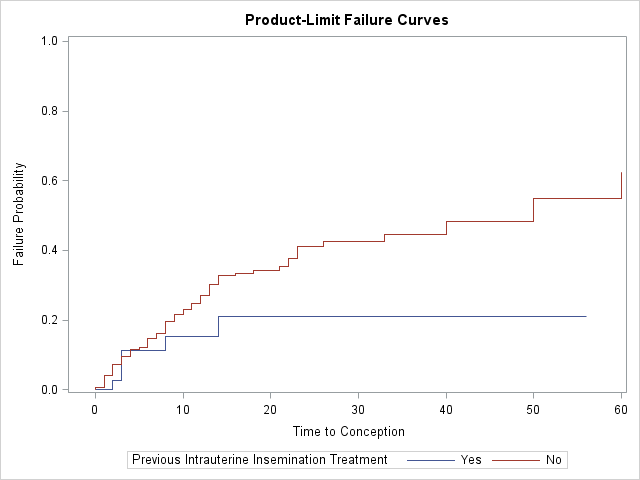


**Figure S-11.** **Cumulative probability of conception resulting in live birth by whether the woman previously received treatment with intrauterine insemination. (Kaplan-Meier curves)**


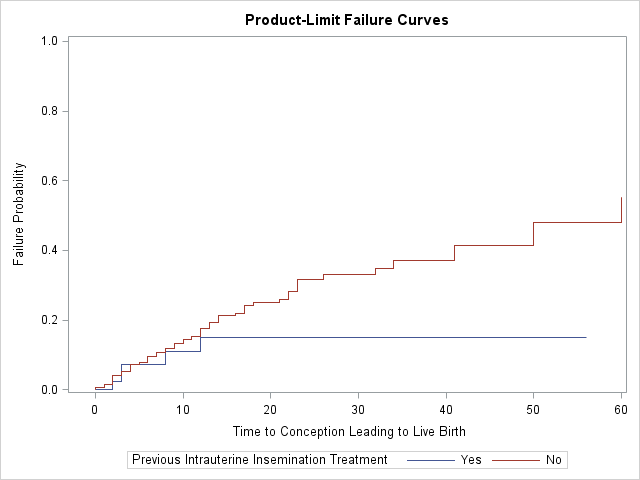


**Figure S-12.** **Cumulative probability of conception by treating physician. (Kaplan-Meier curves)**


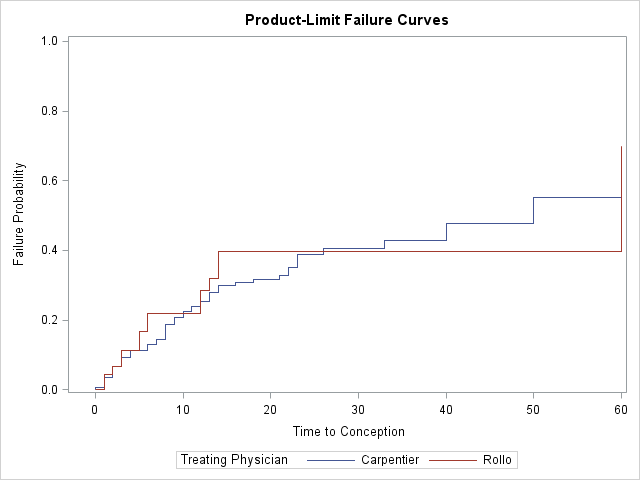


**Figure S-13.** **Cumulative probability of conception resulting in live birth by treating physician. (Kaplan-Meier curves)**


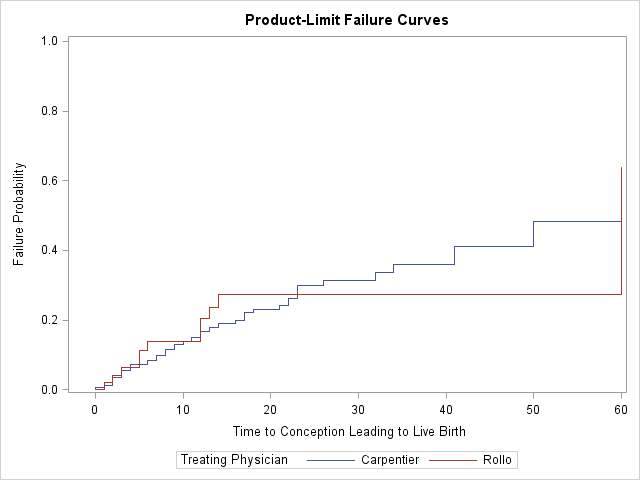


**Figure S-14.** **Cumulative probability of conception by type of fertility cycle charting. (Kaplan-Meier curves)**


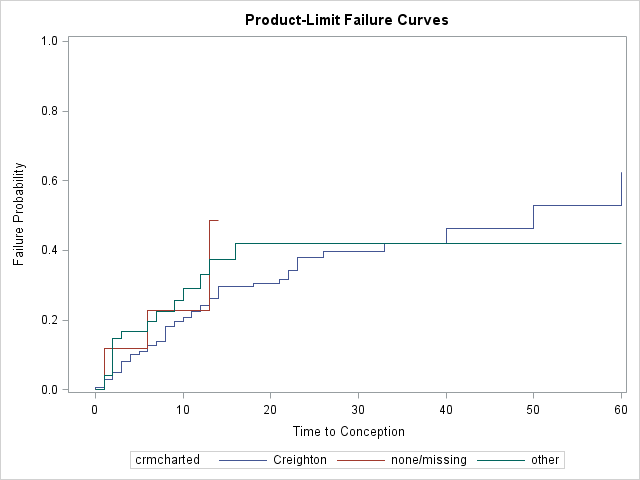


**Figure S-15.** **Cumulative probability of conception leading to live birth by type of fertility cycle charting. (Kaplan-Meier curves)**


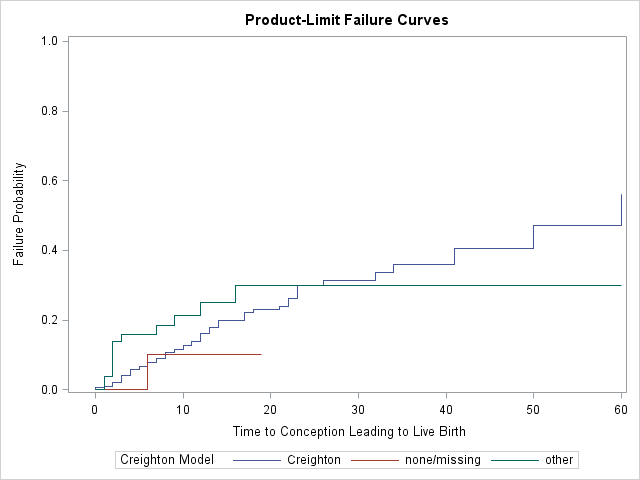


**Figure S-16.** **Cumulative probability of conception by Body Mass Index (Kaplan-Meier curves).**


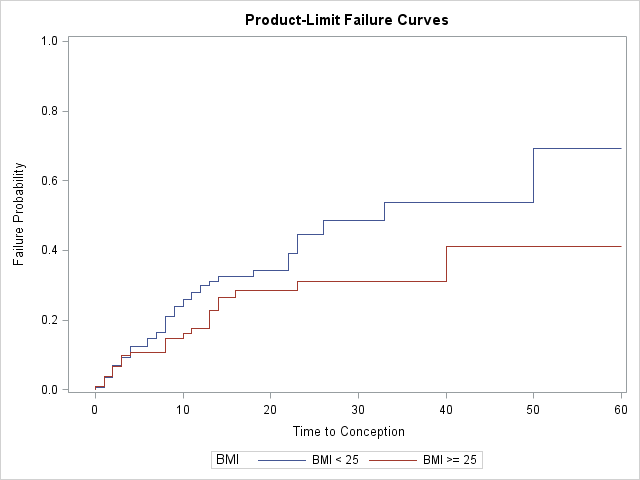


**Figure S-17.** **Cumulative probability of conception by time of entry to NPT treatment (Tertiles of start date; Kaplan-Meier curves).**


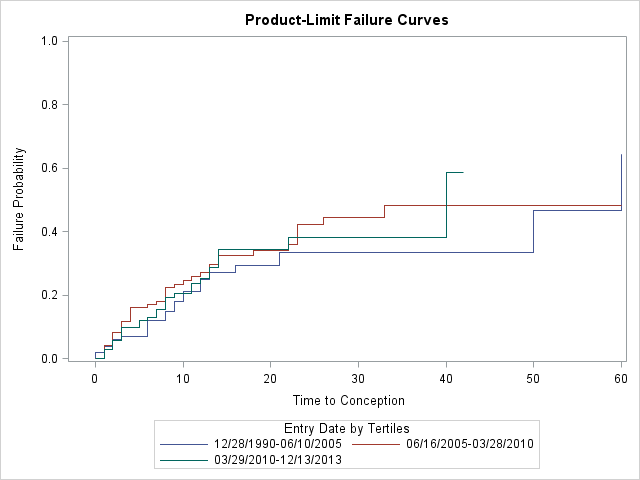


**Figure S-18.** **Cumulative probability of conception leading to live birth by time of entry to NPT treatment (Tertiles of start date; Kaplan-Meier curves).**


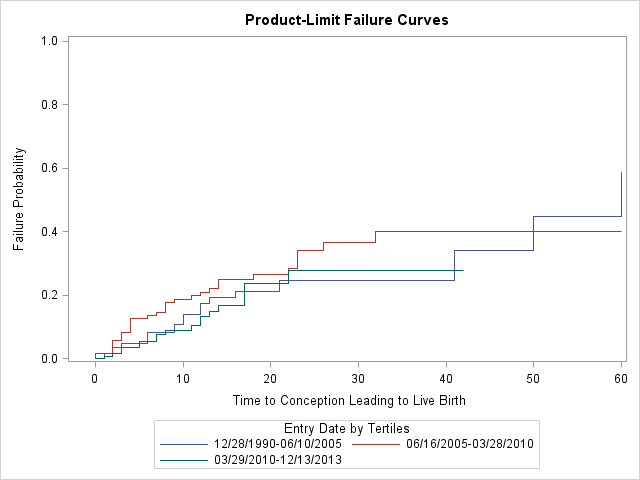

Supplement: Supplementary file 1 — Additional file 1. [file 12884_2021_3946_MOESM1_ESM.zip › RRM-NewEngland-APPENDIX-v36R2.docx]
