# Supplementary figures and images for: Restorative reproductive medicine for infertility in two family medicine clinics in New England, an observational study
Source: BMC Pregnancy Childbirth. 2021 Jul 7;21:495. doi: 10.1186/s12884-021-03946-8 (PMC8265110; doi:10.1186/s12884-021-03946-8)

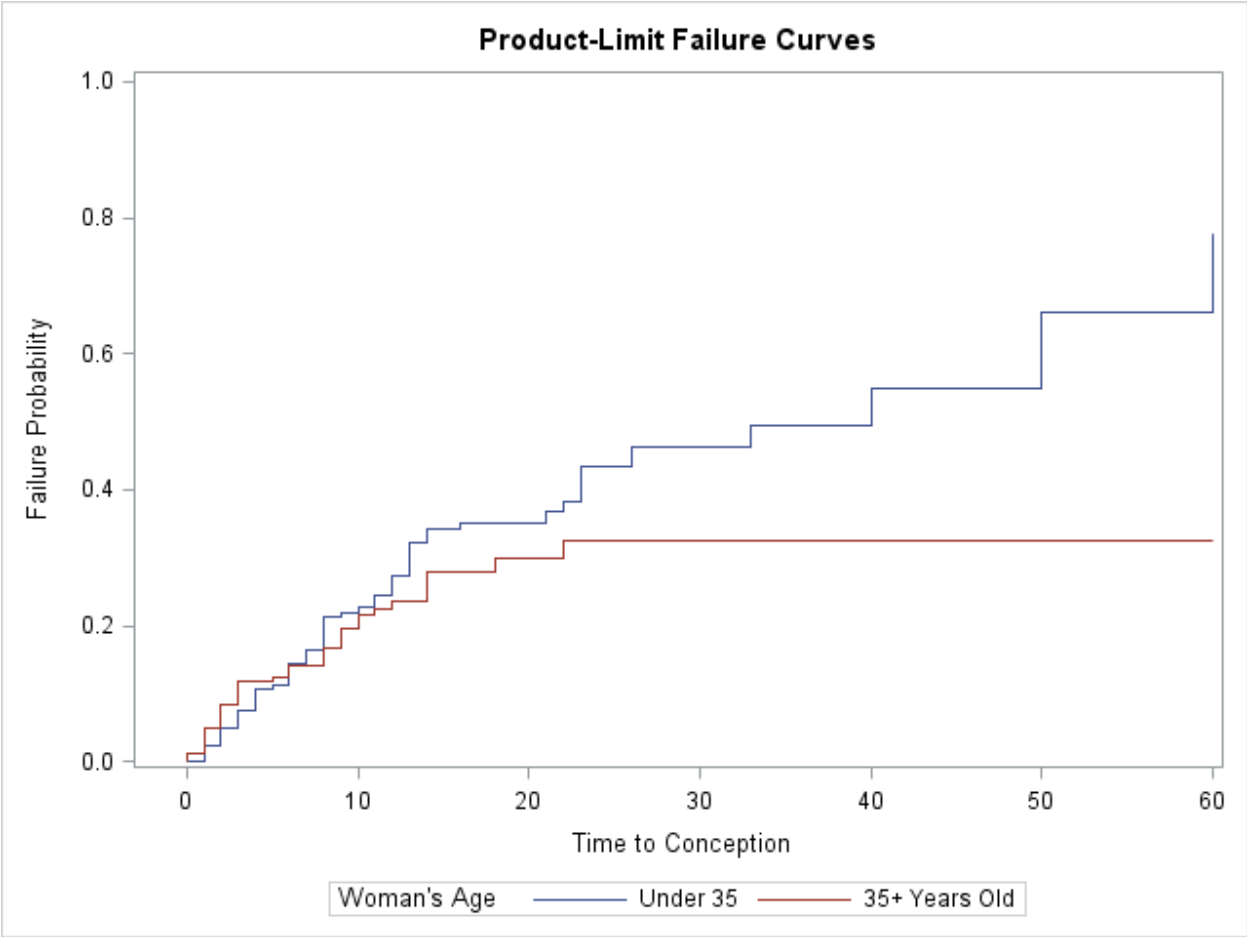

Supplement: Supplementary file 1 — Additional file 1. [file 12884_2021_3946_MOESM1_ESM.zip › RRM-NewEngland-APPENDIX-v36R2_Fig0001.pdf]

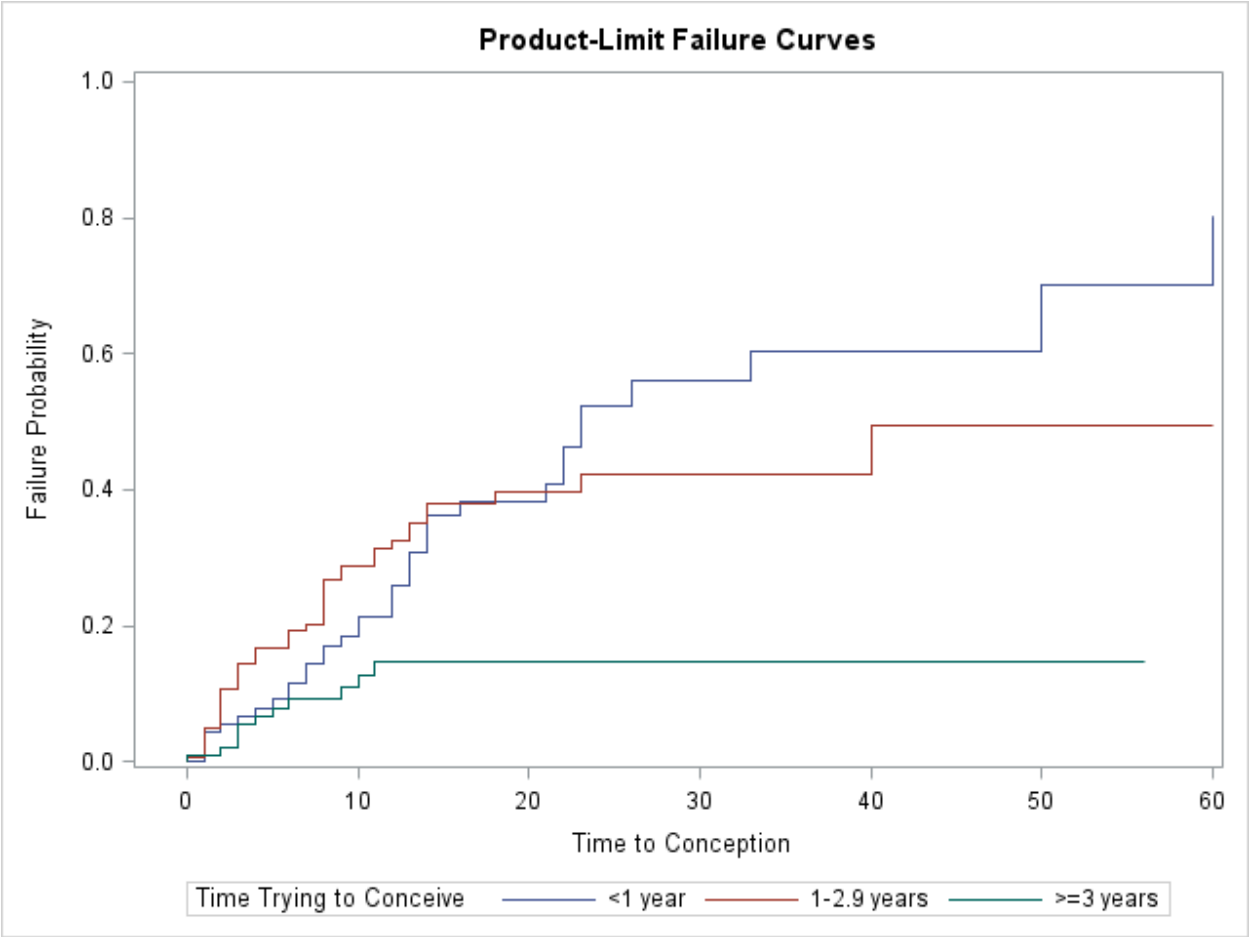

Supplement: Supplementary file 1 — Additional file 1. [file 12884_2021_3946_MOESM1_ESM.zip › RRM-NewEngland-APPENDIX-v36R2_Fig0002.pdf]

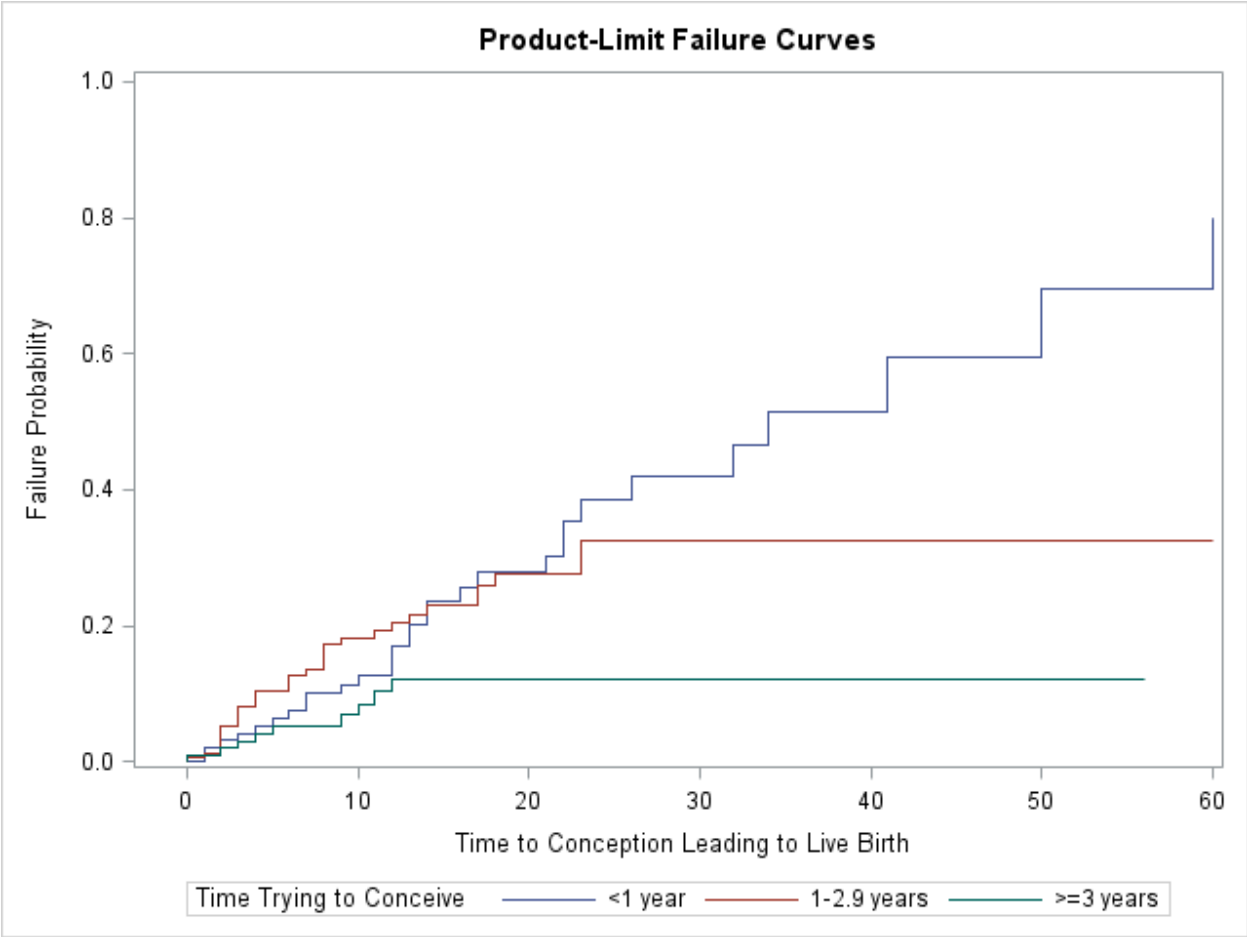

Supplement: Supplementary file 1 — Additional file 1. [file 12884_2021_3946_MOESM1_ESM.zip › RRM-NewEngland-APPENDIX-v36R2_Fig0003.pdf]

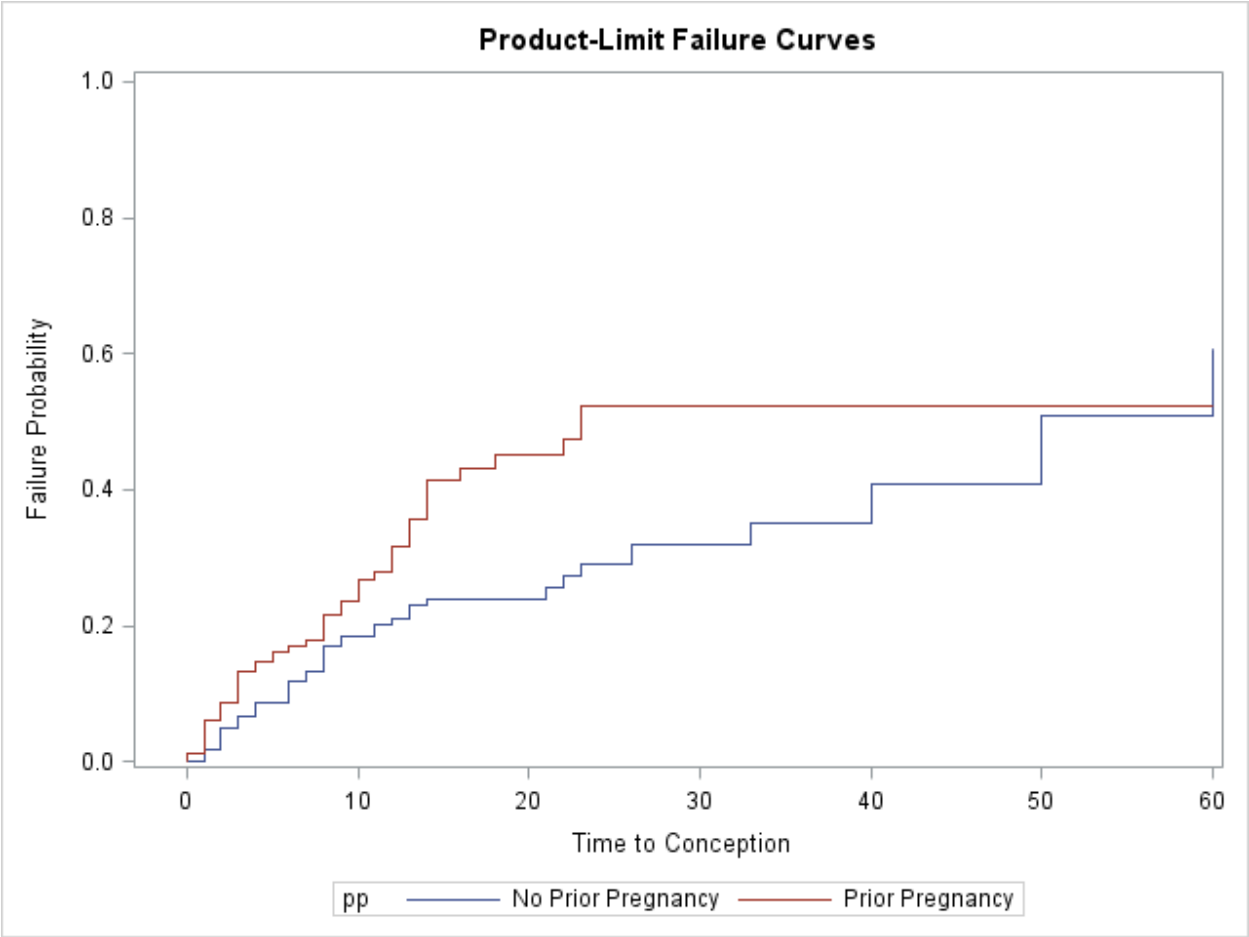

Supplement: Supplementary file 1 — Additional file 1. [file 12884_2021_3946_MOESM1_ESM.zip › RRM-NewEngland-APPENDIX-v36R2_Fig0004.pdf]

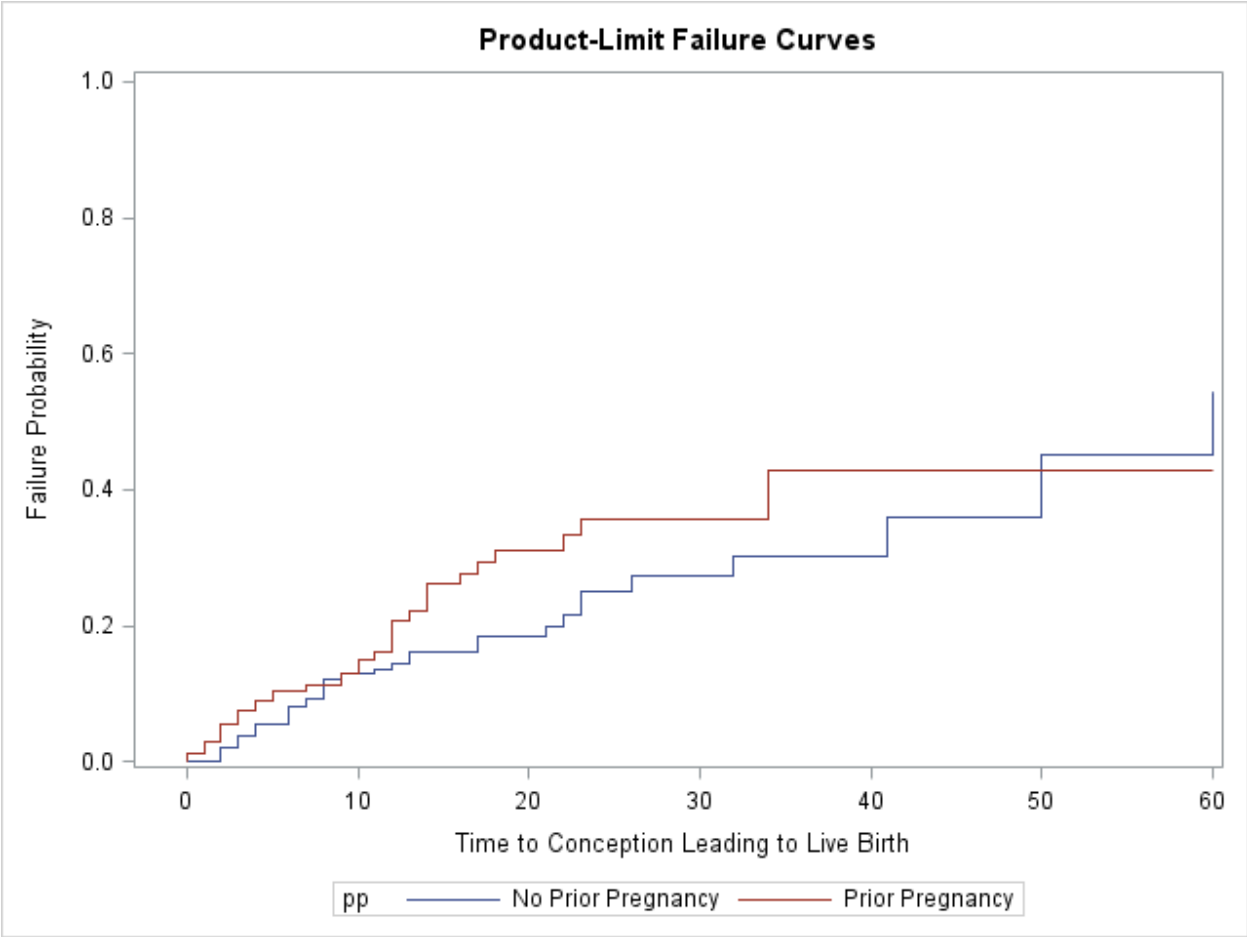

Supplement: Supplementary file 1 — Additional file 1. [file 12884_2021_3946_MOESM1_ESM.zip › RRM-NewEngland-APPENDIX-v36R2_Fig0005.pdf]

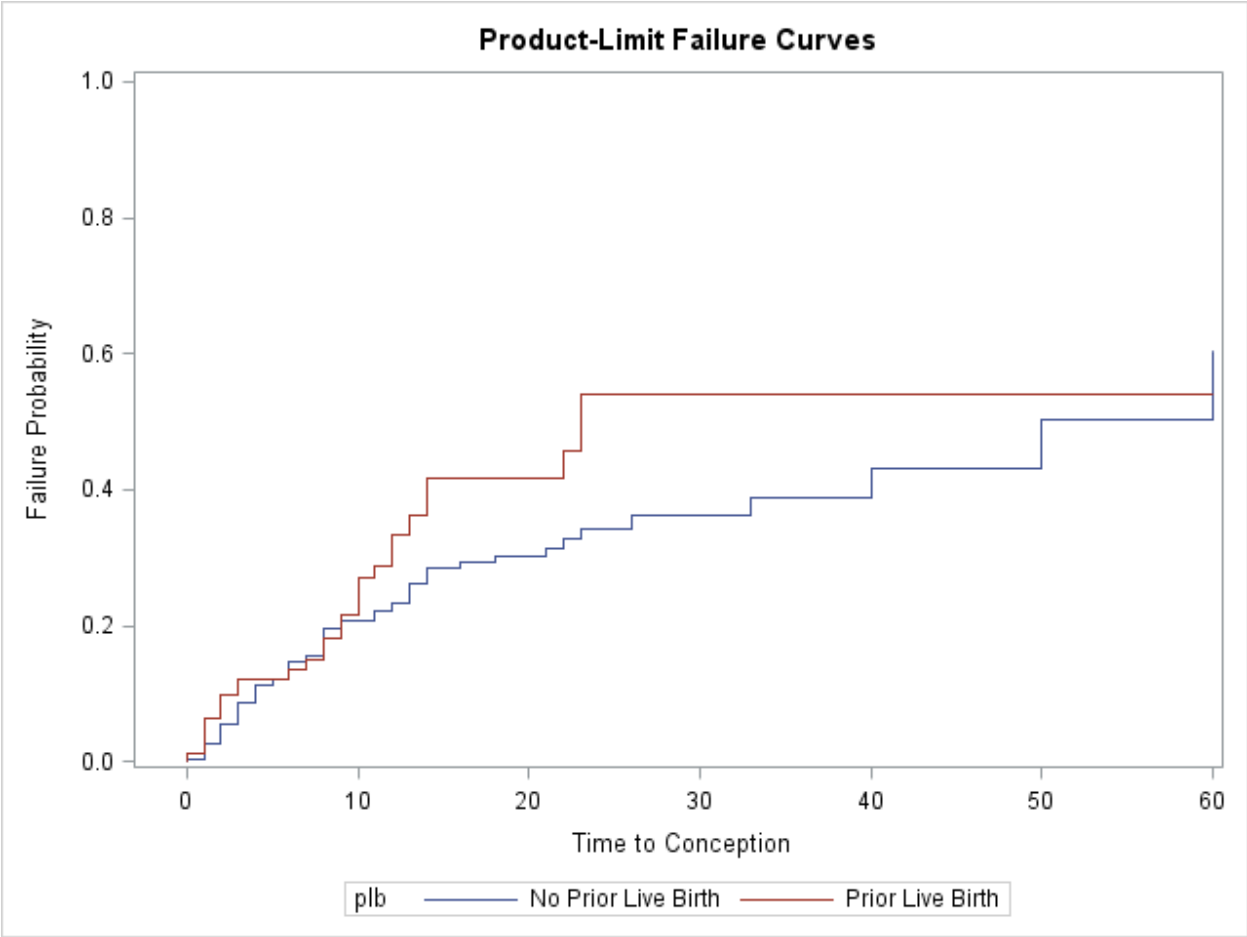

Supplement: Supplementary file 1 — Additional file 1. [file 12884_2021_3946_MOESM1_ESM.zip › RRM-NewEngland-APPENDIX-v36R2_Fig0006.pdf]

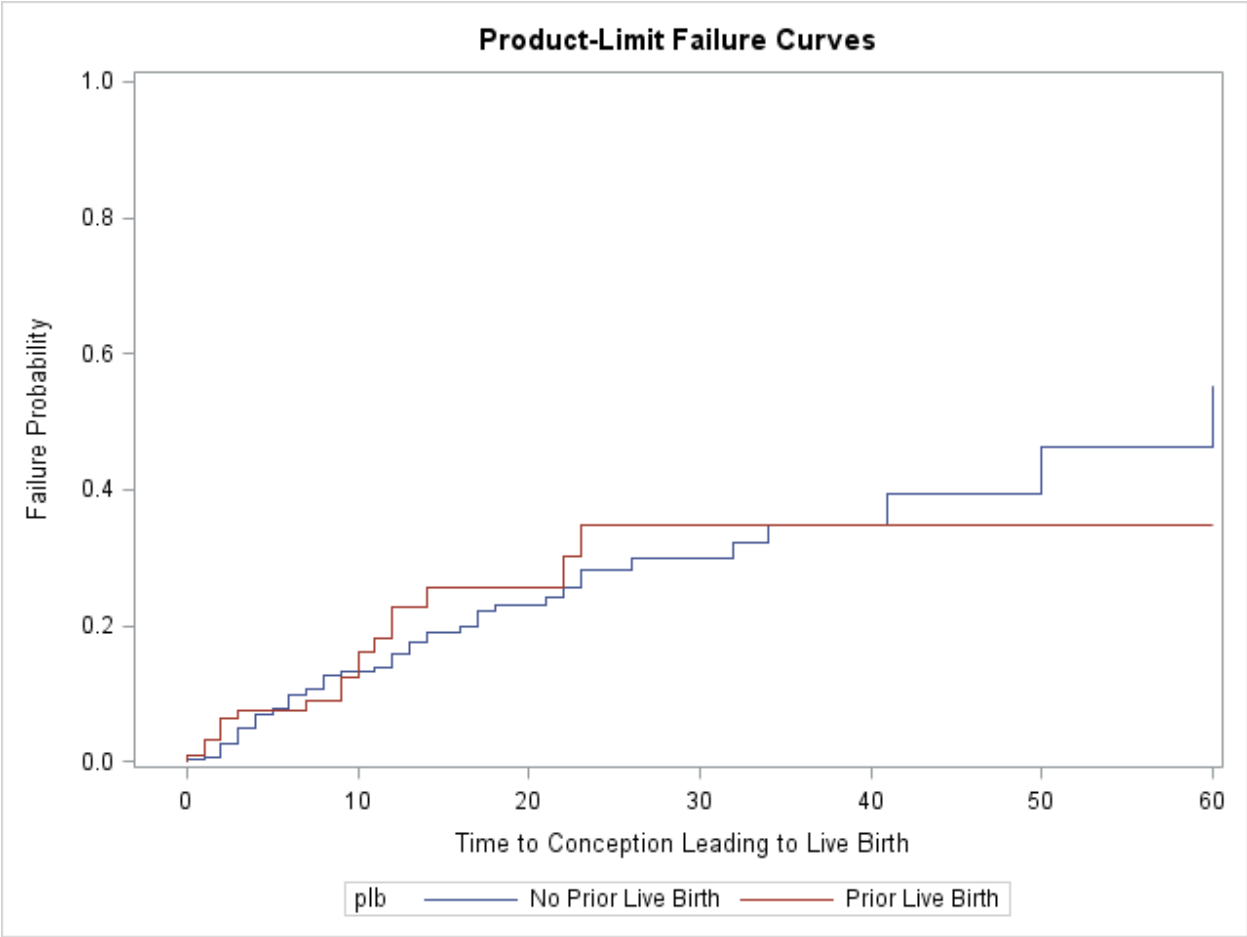

Supplement: Supplementary file 1 — Additional file 1. [file 12884_2021_3946_MOESM1_ESM.zip › RRM-NewEngland-APPENDIX-v36R2_Fig0007.pdf]

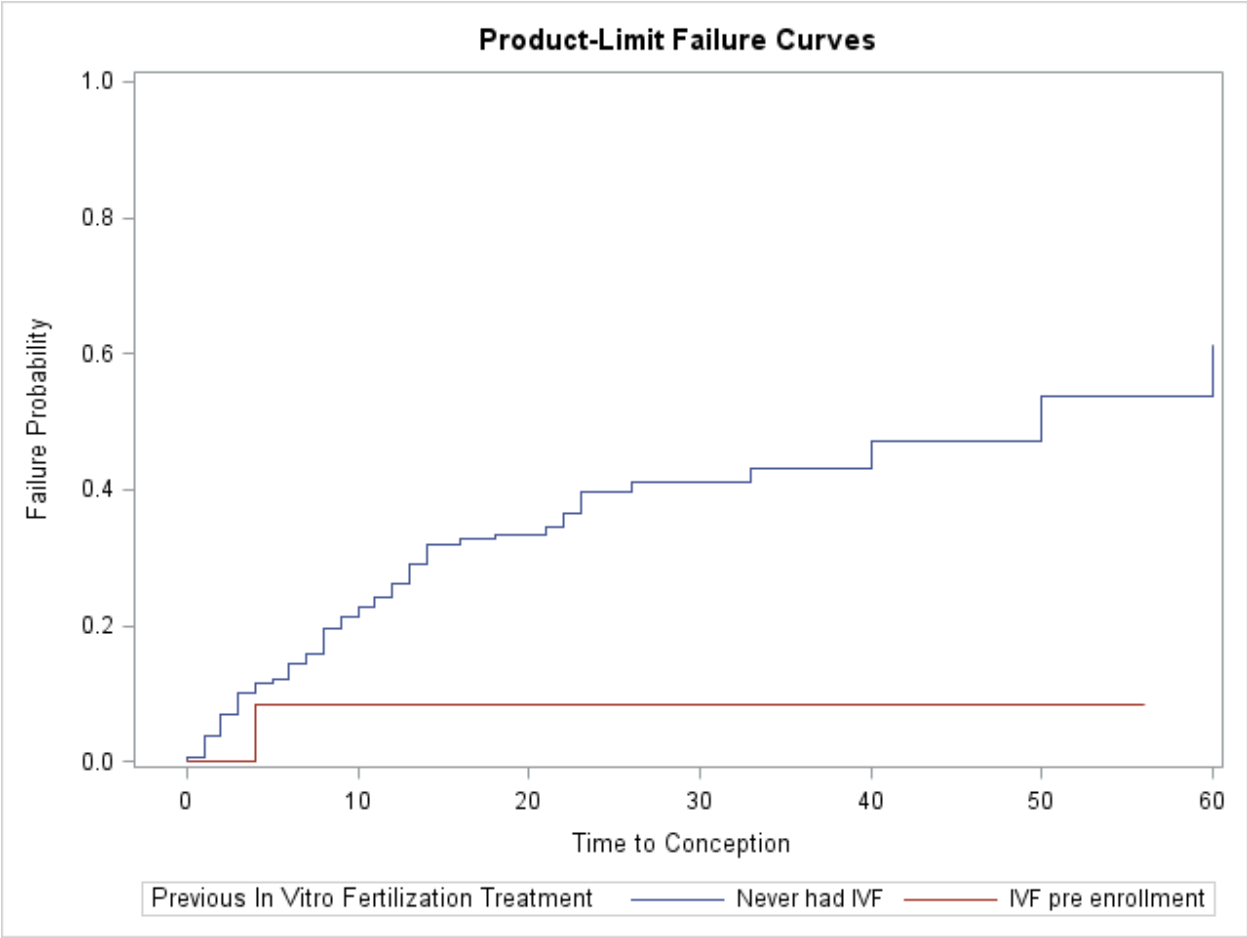

Supplement: Supplementary file 1 — Additional file 1. [file 12884_2021_3946_MOESM1_ESM.zip › RRM-NewEngland-APPENDIX-v36R2_Fig0008.pdf]

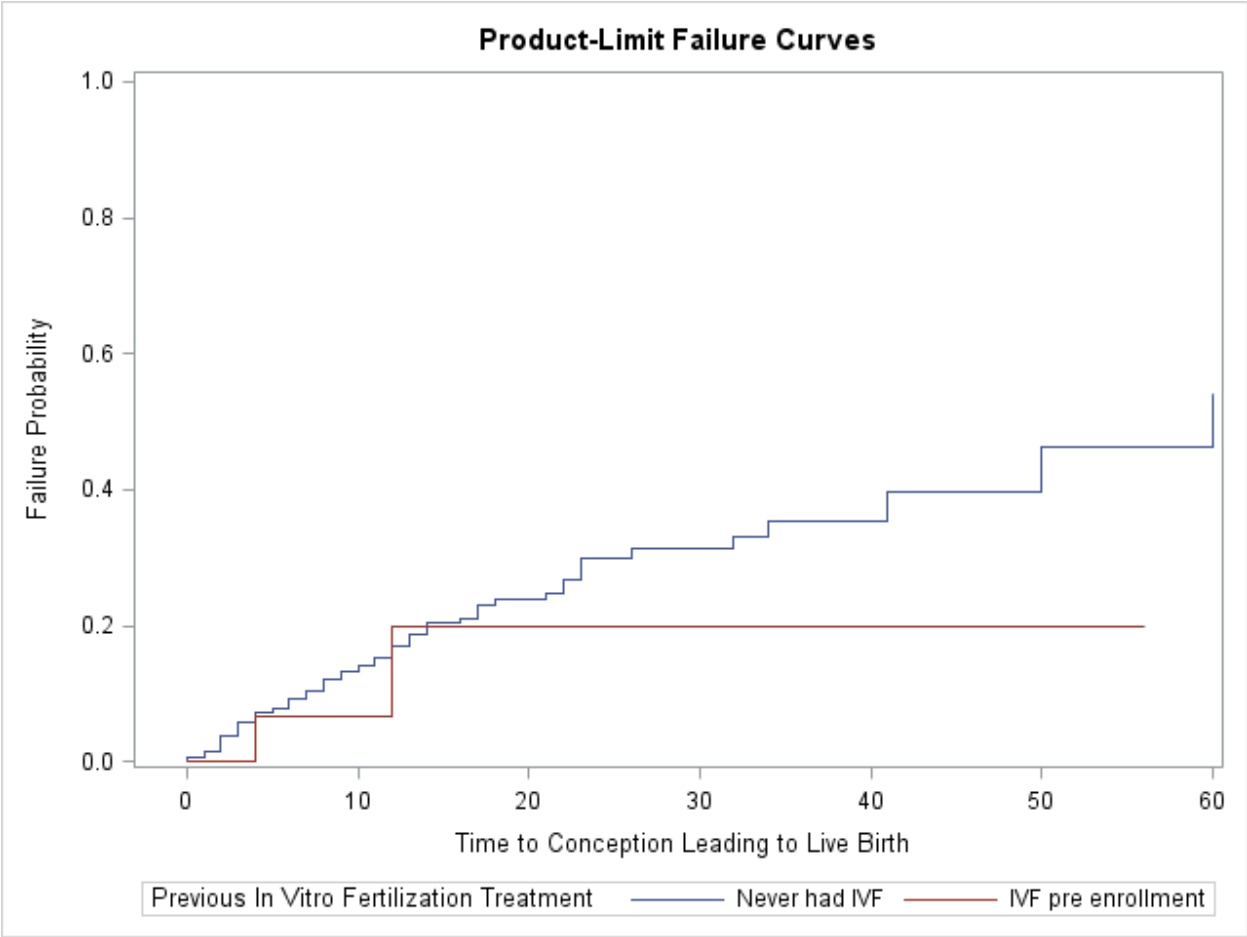

Supplement: Supplementary file 1 — Additional file 1. [file 12884_2021_3946_MOESM1_ESM.zip › RRM-NewEngland-APPENDIX-v36R2_Fig0009.pdf]

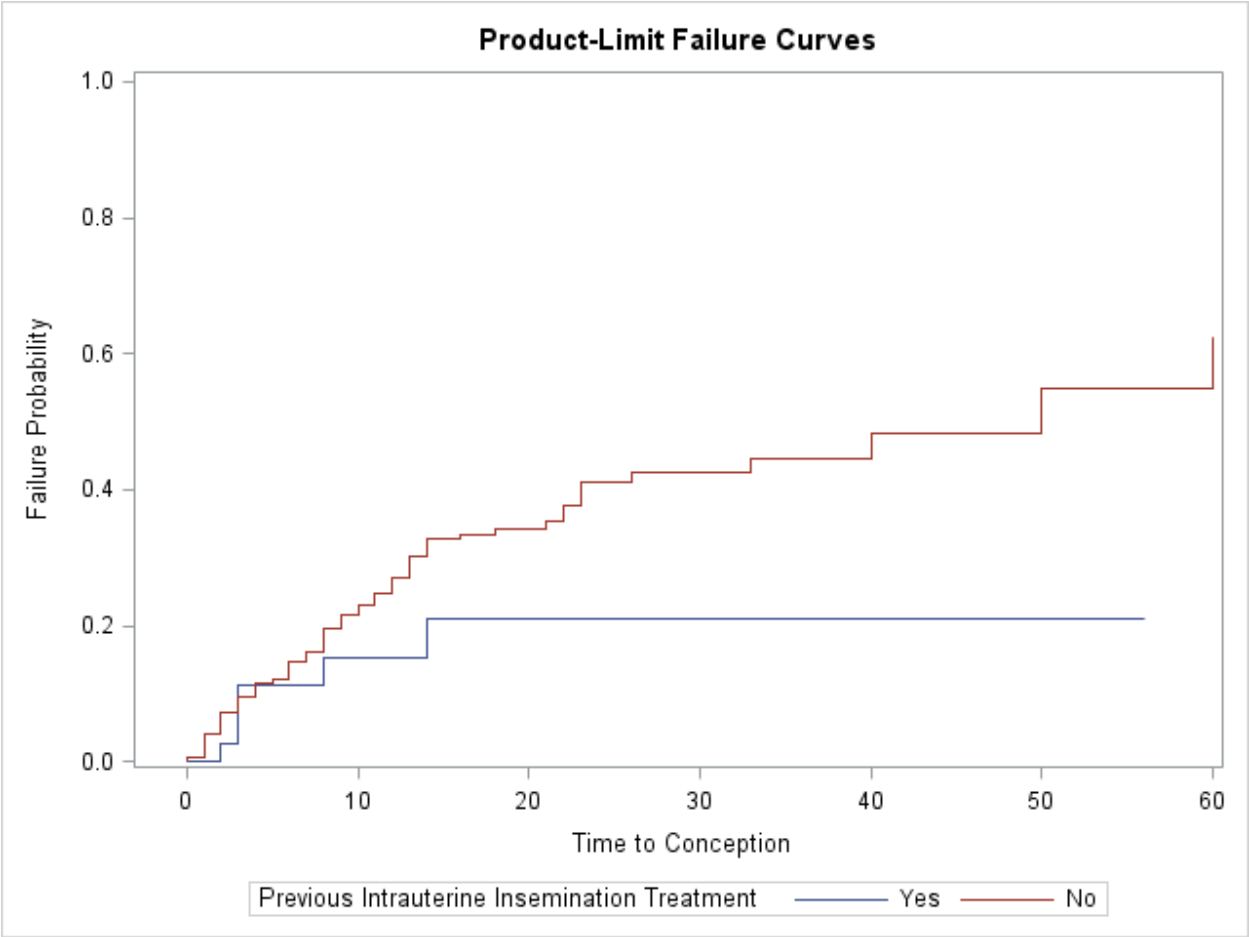

Supplement: Supplementary file 1 — Additional file 1. [file 12884_2021_3946_MOESM1_ESM.zip › RRM-NewEngland-APPENDIX-v36R2_Fig0010.pdf]

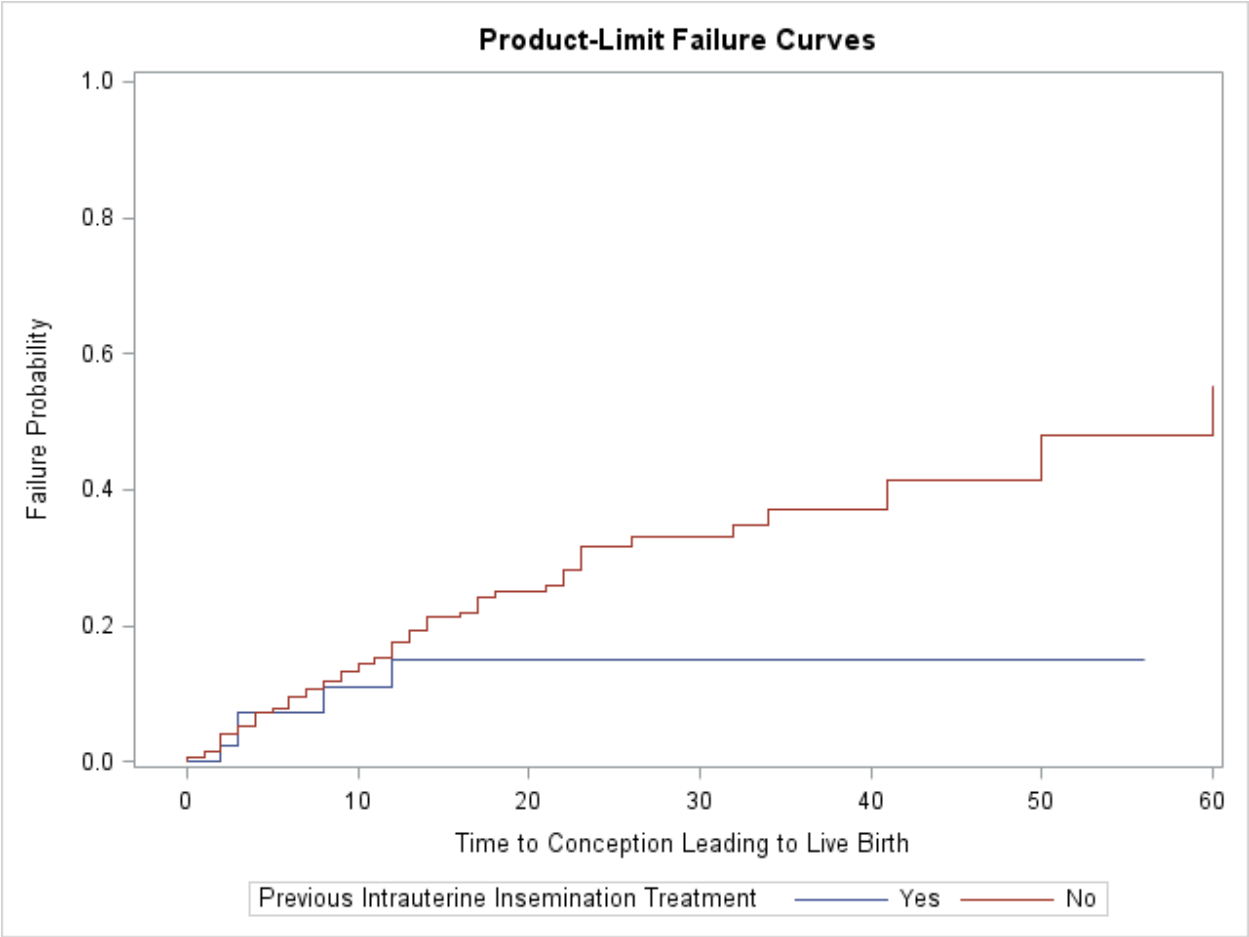

Supplement: Supplementary file 1 — Additional file 1. [file 12884_2021_3946_MOESM1_ESM.zip › RRM-NewEngland-APPENDIX-v36R2_Fig0011.pdf]

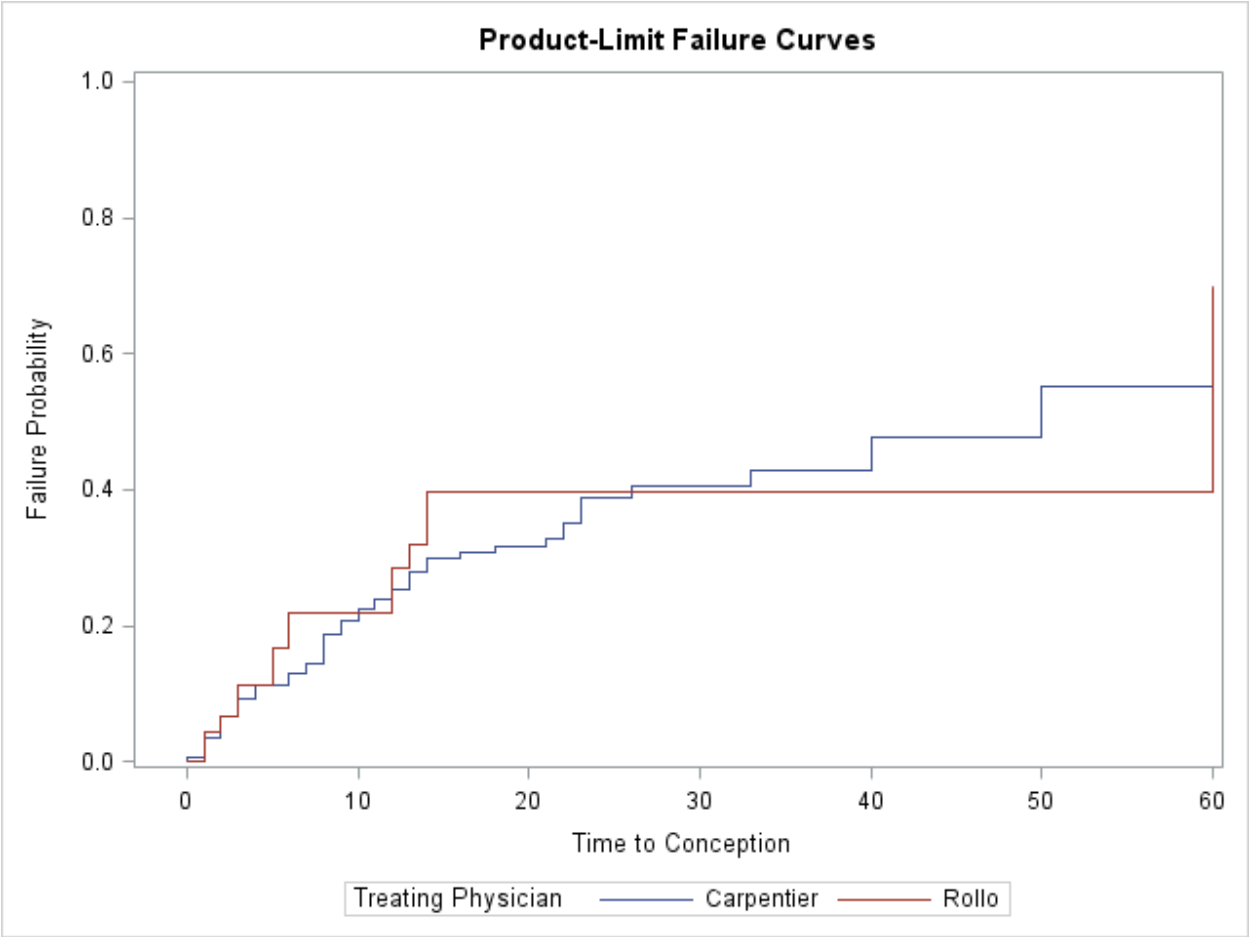

Supplement: Supplementary file 1 — Additional file 1. [file 12884_2021_3946_MOESM1_ESM.zip › RRM-NewEngland-APPENDIX-v36R2_Fig0012.pdf]

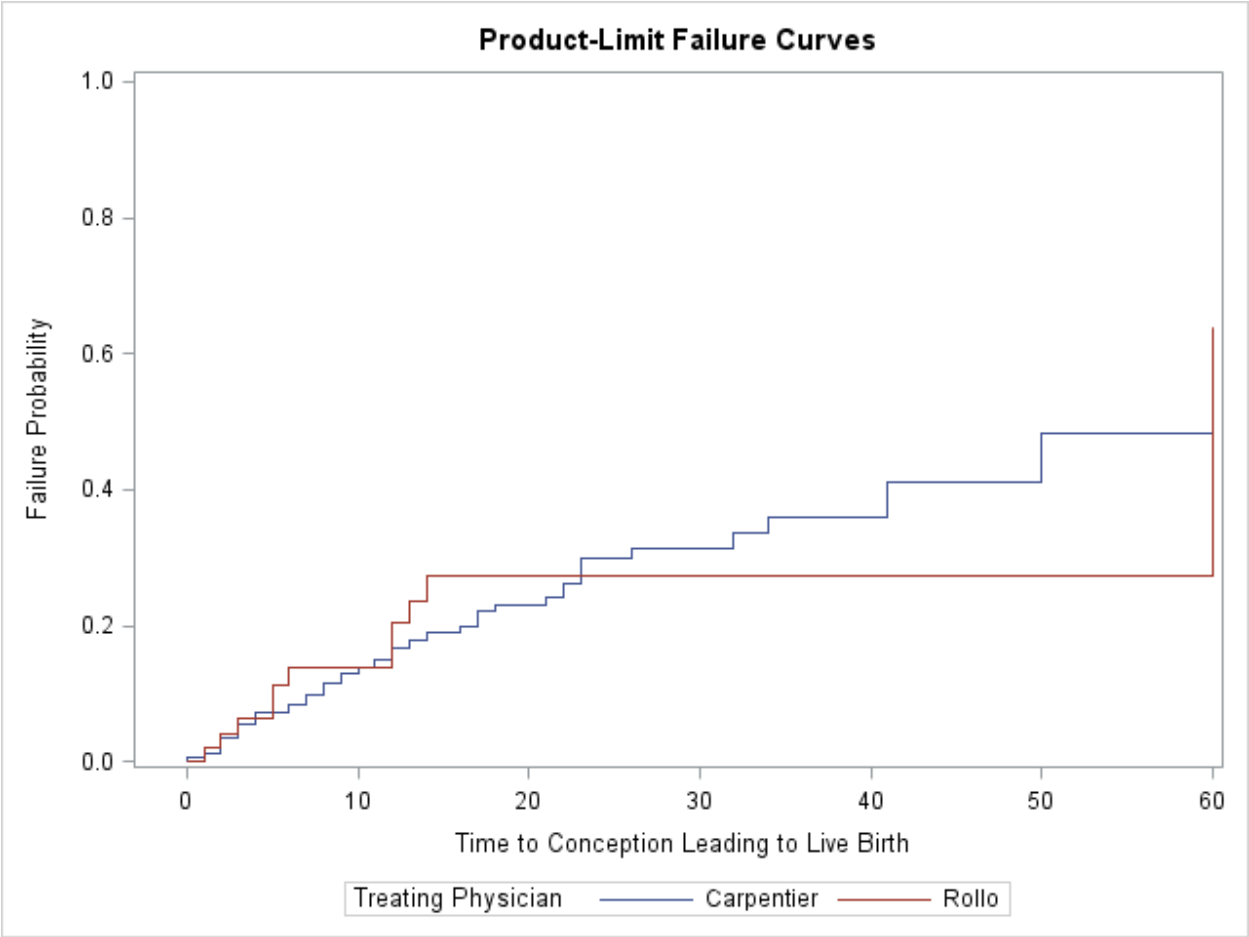

Supplement: Supplementary file 1 — Additional file 1. [file 12884_2021_3946_MOESM1_ESM.zip › RRM-NewEngland-APPENDIX-v36R2_Fig0013.pdf]

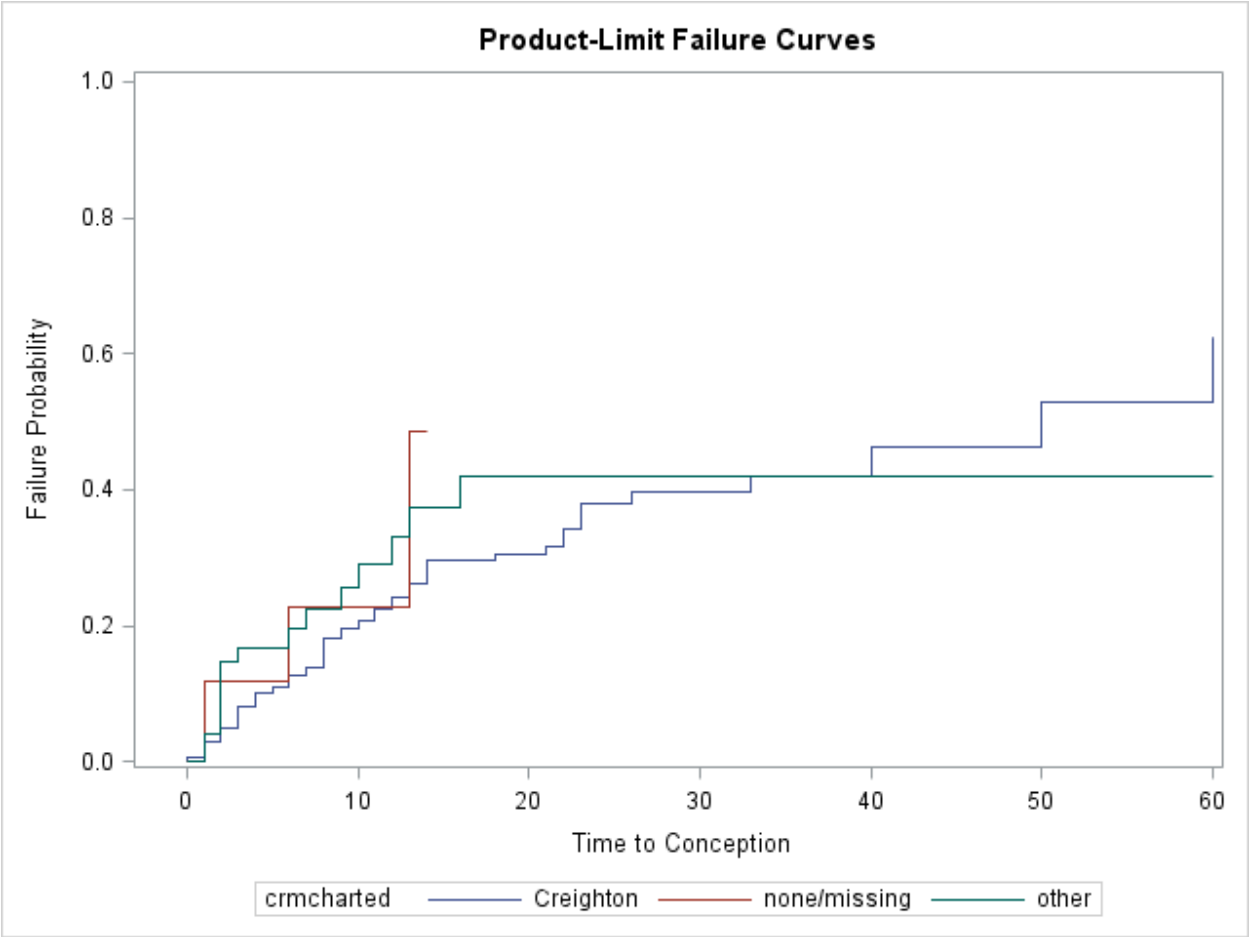

Supplement: Supplementary file 1 — Additional file 1. [file 12884_2021_3946_MOESM1_ESM.zip › RRM-NewEngland-APPENDIX-v36R2_Fig0014.pdf]

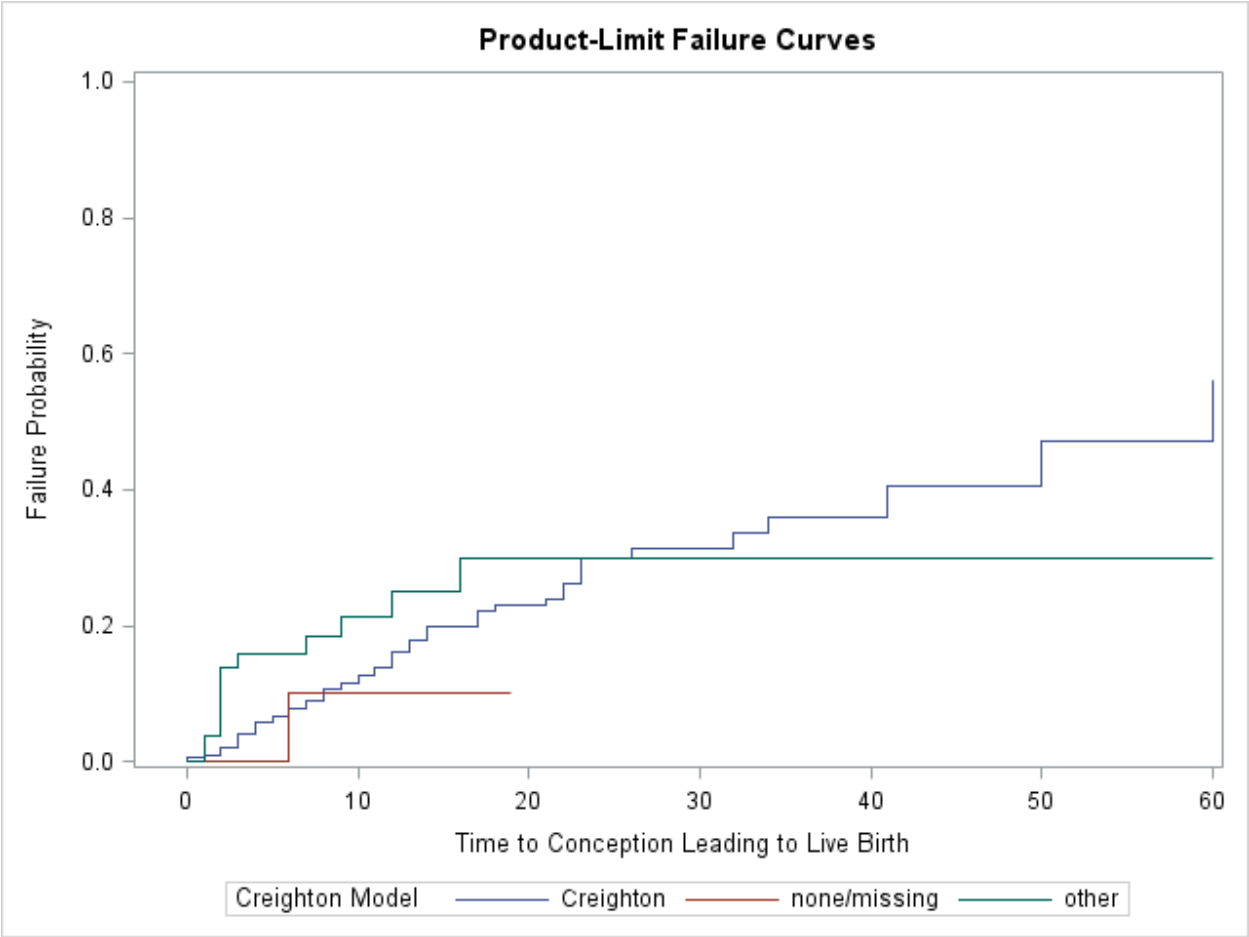

Supplement: Supplementary file 1 — Additional file 1. [file 12884_2021_3946_MOESM1_ESM.zip › RRM-NewEngland-APPENDIX-v36R2_Fig0015.pdf]

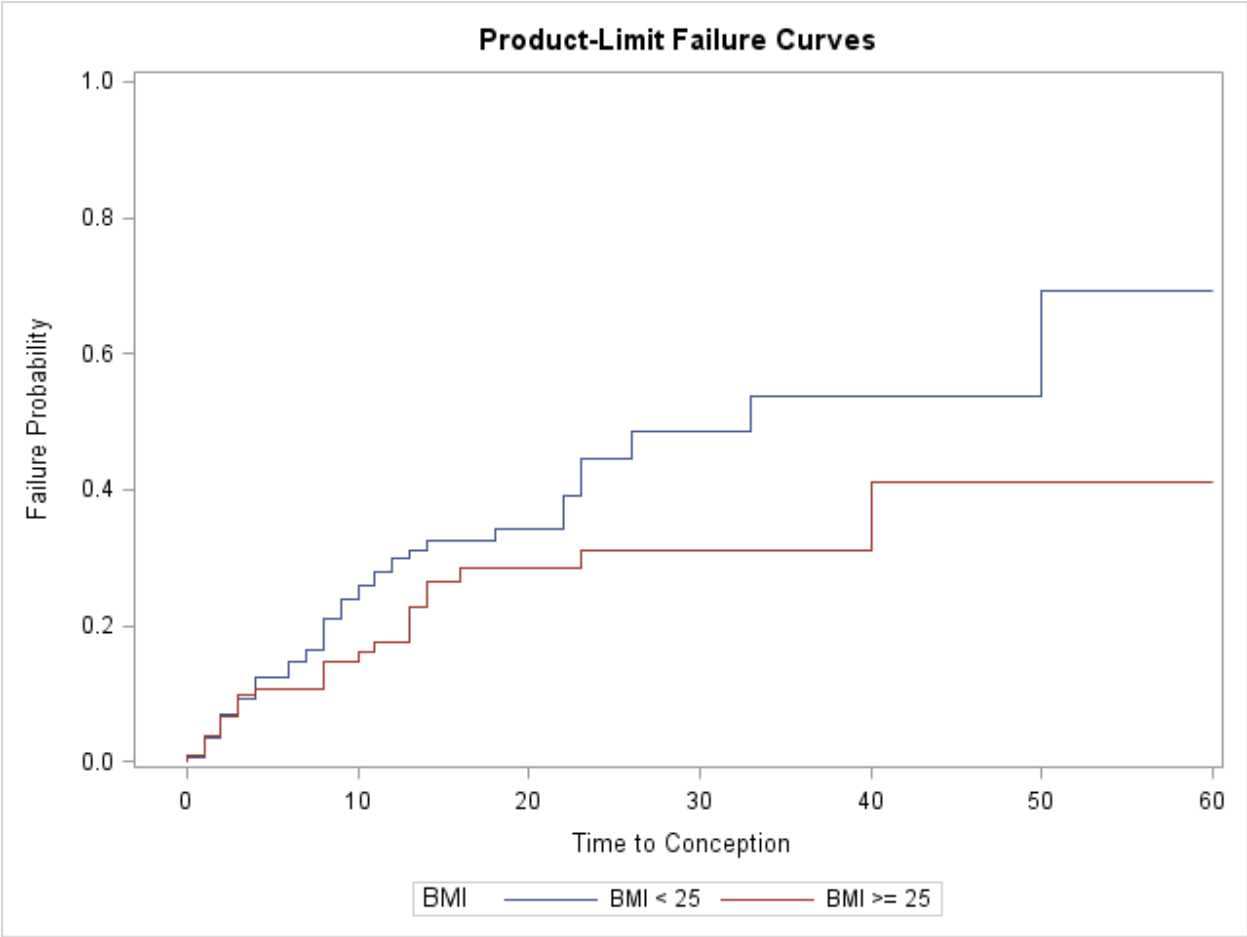

Supplement: Supplementary file 1 — Additional file 1. [file 12884_2021_3946_MOESM1_ESM.zip › RRM-NewEngland-APPENDIX-v36R2_Fig0016.pdf]

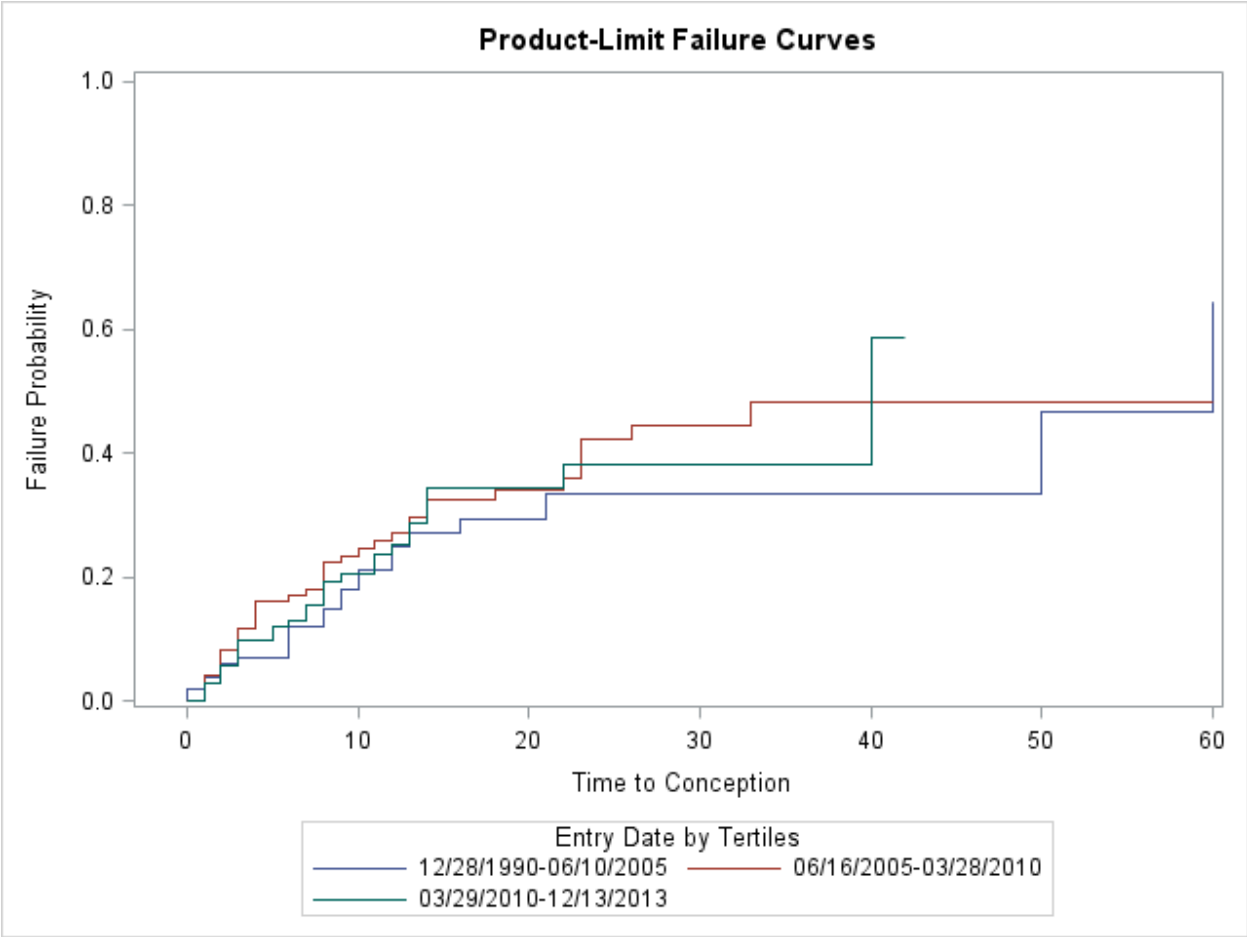

Supplement: Supplementary file 1 — Additional file 1. [file 12884_2021_3946_MOESM1_ESM.zip › RRM-NewEngland-APPENDIX-v36R2_Fig0017.pdf]

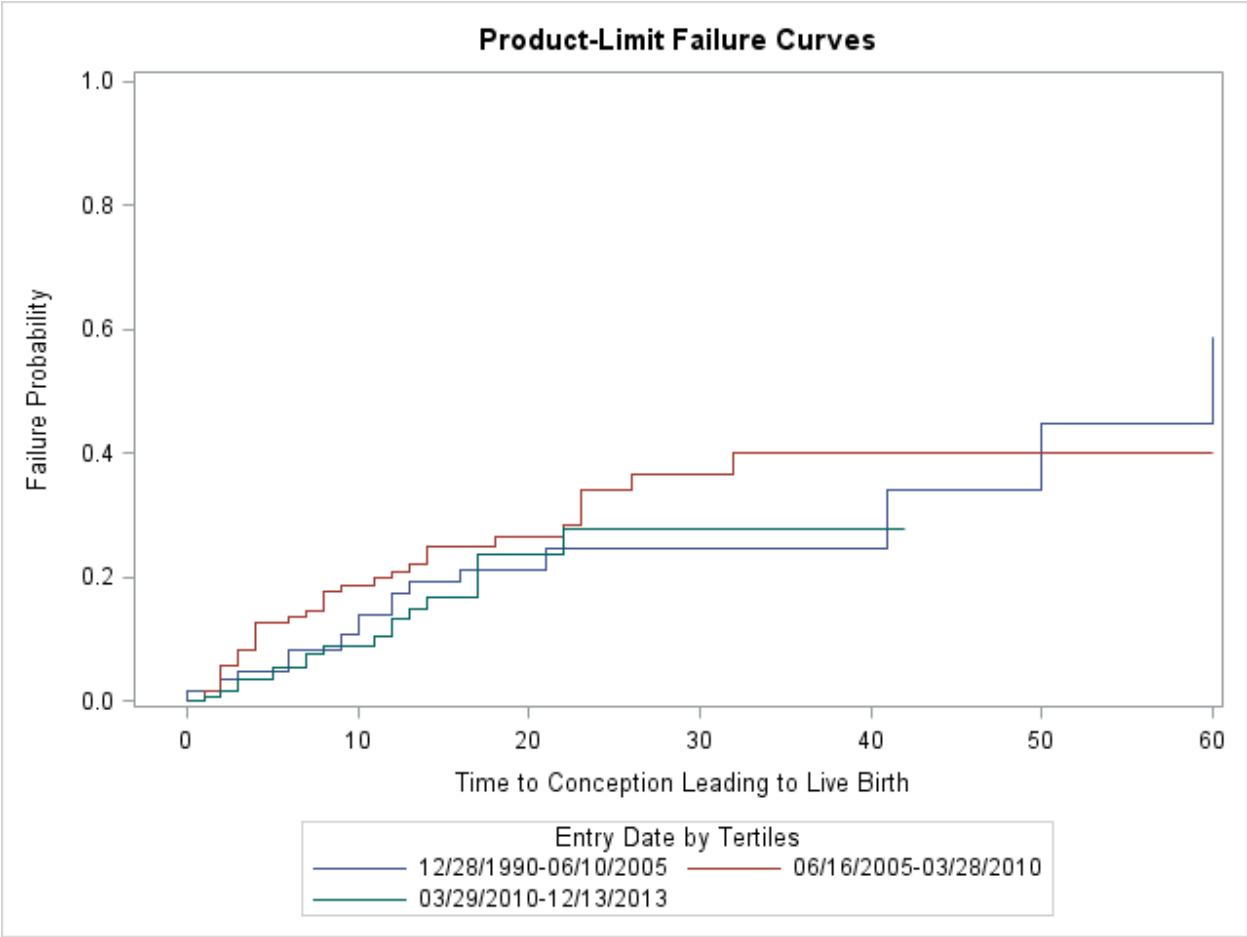

Supplement: Supplementary file 1 — Additional file 1. [file 12884_2021_3946_MOESM1_ESM.zip › RRM-NewEngland-APPENDIX-v36R2_Fig0018.pdf]

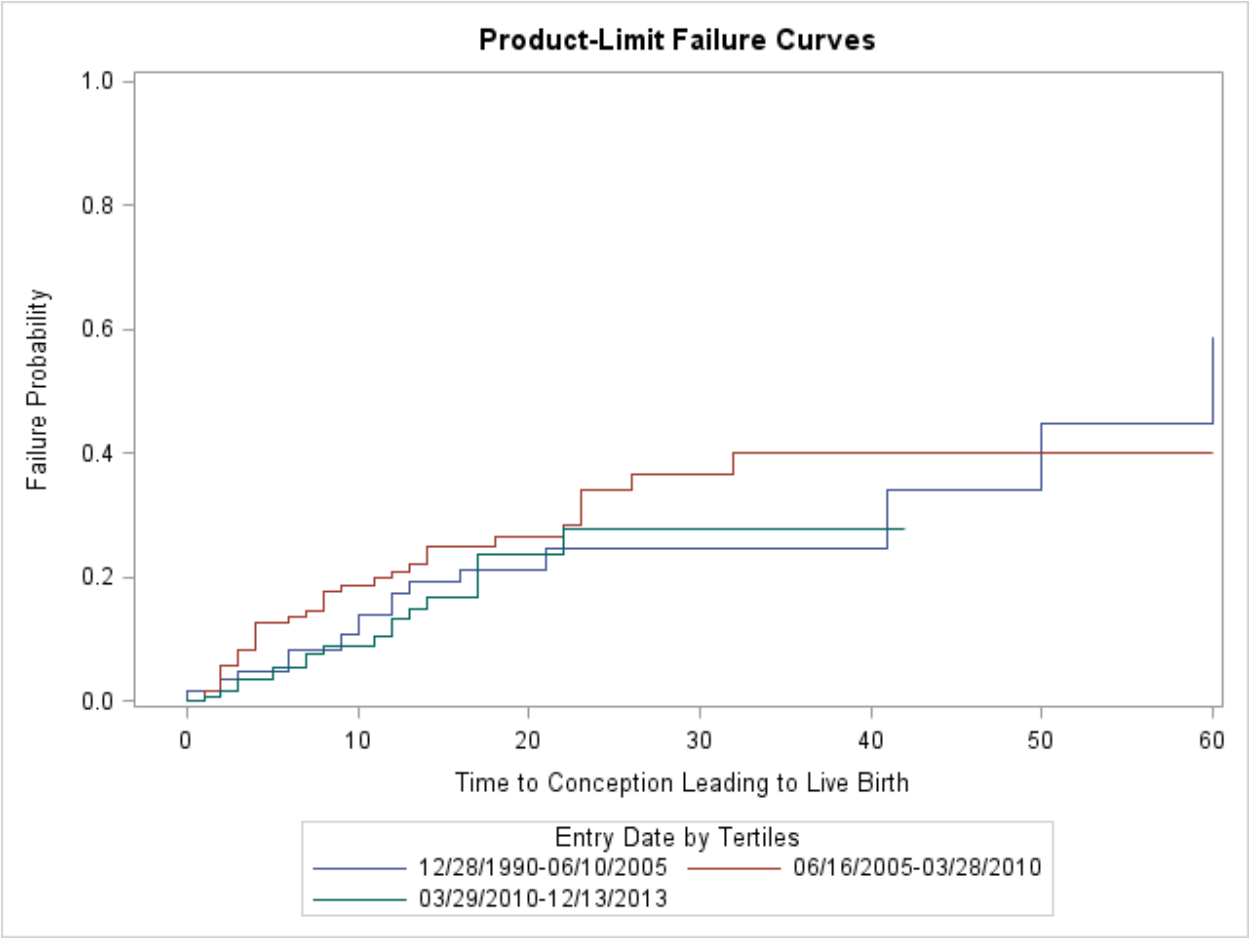

Supplement: Supplementary file 1 — Additional file 1. [file 12884_2021_3946_MOESM1_ESM.zip › RRM-NewEngland-APPENDIX-v36R2_UNFIG0001.pdf]
